# Investigations on the polymorphism of K_4_CaSi_6_O_15_ at elevated temperatures

**DOI:** 10.1111/jace.19310

**Published:** 2023-07-13

**Authors:** Hang Liu, Volker Kahlenberg, Hannes Krüger, Edgar Dachs, Artur Benisek

**Affiliations:** ^1^ Institute of Mineralogy and Petrography University of Innsbruck Innsbruck Austria; ^2^ Department of Materials Science and Physics University of Salzburg Salzburg Austria

**Keywords:** crystal structure, heat capacity, K_2_O‐CaO‐SiO_2_ system, K_4_CaSi_6_O_15_, phase transition

## Abstract

In the present study, single crystals and polycrystalline material of K_4_CaSi_6_O_15_ were prepared from solid‐state reactions between stoichiometric mixtures of the corresponding oxides/carbonates. Heat capacity (*C*_p_) measurements above room temperature using a differential scanning calorimeter indicated that two thermal effects occurred at approximately *T*_1_ = 462 K and *T*_2_ = 667 K, indicating the presence of structural phase transitions. The standard third‐law entropy of K_4_CaSi_6_O_15_ was determined from low‐temperature *C*_p_’s measured by relaxation calorimetry using a Physical Properties Measurement System and amounts to *S*°(298K) = 524.3 ± 3.7 J·mol^−1^·K^−1^. For the 1^st^ transition, the enthalpy change Δ*H* ^tr1^ = 1.48 kJ·mol and the entropy change Δ*S* ^tr1^ = 3.25 J·mol^−1^·K^−1^, whereas Δ*H* ^tr2^ = 3.33 kJ·mol^−1^ and Δ*S* ^tr2^ = 5.23 J·mol^−1^·K^−1^ were determined for the 2^nd^ transition. The compound was further characterized by in‐situ single‐crystal X‐ray diffraction between ambient temperature and 1063 K. At 773 K, the high‐temperature phase stable above *T*_2_ has the following basic crystallographic data: monoclinic symmetry, space group *P*2_1_/*c*, *a* = 6.9469(4) Å, *b* = 9.2340(5) Å, *c* = 12.2954(6) Å, β = 93.639(3)°, *V* = 787.13(7) Å^3^, *Z* = 2. It belongs to the group of interrupted framework silicates and is based on tertiary (Q^3^‐type) [SiO_4_]‐tetrahedra. Together with the octahedrally coordinated Ca‐cations, a three‐dimensional mixed polyhedral network structure is formed, in which the remaining K‐ions provide charge balance by occupying voids within the net. The intermediate temperature modification stable between *T*_1_ and *T*_2_ shows a (3+2)‐dimensional incommensurately modulated structure that is characterized by the following q‐vectors: q_1_ = (0.057, 0.172, 0.379), q_2_ = (‐0.057, 0.172, ‐0.379). The crystal structures of the high‐ and the previously studied ambient temperature polymorph (space group *Pc*) are topologically equivalent and show a group‐subgroup relationship. The index of the low‐ in the high‐symmetry group is six and involves both, losses in translation as well as point group symmetry. The distortion is based on shifts of the different atom species and tilts of the 4‐ and 6‐fold coordination polyhedra. Actually, for some of the oxygen atoms, the displacements exceed 0.5 Å. A more detailed analysis of the distortions relating to both structures has been performed using mode analysis, which revealed that the primary distortion mode transforms according to the Λ_1_ irreducible representation of *P*2_1_/*c*. However, other modes with smaller distortion amplitudes are also involved.

## 1 INTRODUCTION

In the context of the increasing severity of the greenhouse impact, carbon neutrality has become an urgent demand. Renewable and carbon‐neutral energy sources such as biomass or agricultural waste have seen increased market shares during the past decade. Their intensified usage can be an efficient policy tool for environmentally sustainable development.^1^, ^2^ As potassium calcium silicates play an important role in residual materials, including ashes from biomass combustion, but also in steelmaking slags or residues of the oil‐shale industry,^3^, ^4^, ^5^, ^6^, ^7^, ^8^, ^9^, ^10^, ^11^, ^12^, ^13^, ^14^ the K_2_O‐CaO‐SiO_2_ (KCS) oxide system has been in the focus of attention for a long time. To reduce or even better prevent serious problems in the combustion chambers such as slagging, corrosion, or fouling, which can in the worst case lead to a complete shutdown of the biomass energy plant,^15^, ^16^ it is necessary to obtain a better understanding of the chemical and physical properties of these residual materials.

In the case of the K_2_O‐CaO‐SiO_2_ system, experimental phase equilibrium data are difficult to obtain due to the volatility of potassium,^17^ hygroscopicity or high viscosity of the silica‐rich melts. Actually, accurate thermodynamic modeling using the semi‐empirical CALPHAD technique could be very helpful in predicting the behavior of potassium calcium silicates at high temperatures. Nevertheless, for the ternary phases present in the system, their Gibbs energies have to be assessed using results of experimental observations rather than employing “pure binary extensions.”^18^ Therefore, not only the knowledge of all existing binary and ternary phases of this system together with their chemical composition is required, but also information about their thermodynamic properties, such as heat capacity, entropy, enthalpy, and melting points. For polymorphs, in particular, it is necessary to clarify their stability ranges concerning temperature and their structural features as well.

The pioneering work of Morey et al., in 1930,^19^ was relied upon for decades to interpret the phase equilibrium and melt formation in this system. Notably, the validity of several of the phases they described was confirmed in more recent studies (K_8_CaSi_10_O_25_,^20^ K_4_CaSi_3_O_9_,^21^ and K_4_CaSi_6_O_15_ ^22^). However, quite a number of new crystalline compounds not mentioned by them were identified as well (K_2_Ca_2_Si_2_O_7_,^23^ K_2_Ca_3_Si_3_O_10_,^24^ K_2_Ca_6_Si_4_O_15_,^25^ and K_2_CaSi_4_O_10_ ^26^). Among the first group, the presence of K_4_CaSi_6_O_15_ ‐ a stable phase under ambient conditions‐was only very recently confirmed in our own investigations. In a continuation of our studies on this compound, we discovered that it undergoes two structural phase transitions with increasing temperature, a behavior that has not been reported for other established ternary crystalline phases in the K_2_O‐CaO‐SiO_2_ system. The present study discloses the temperatures of the two structural phase transitions and reports a detailed crystallographic description of the high‐temperature modification of K_4_CaSi_6_O_15_ at 773 K including a structural comparison with the ambient temperature polymorph.

## 2 EXPERIMENTAL DETAILS

### 2.1 Synthesis

A sample of K_4_CaSi_6_O_15_ was synthesized following an approach that has been used successfully in a previous study.^22^ CaCO_3_ (Merck, min. 99%), K_2_CO_3_ (Alfa Aesar, 99.997%), and amorphous SiO_2_ (Alfa Aesar, 99.995%) were stoichiometrically weighed after being dried for several hours at 573 K and then homogenized in an agate jar using a planetary ball mill for 45 min with the addition of a small amount of ethanol. After evaporation of the alcohol, the dried educts were pressed into two tablets having a weight of 0.5 g and a diameter of 12 mm. The pellets were encapsulated with platinum foil, placed into a corundum combustion boat, and fired using a horizontal resistance tube furnace at a rate of 50 K/h from room temperature to 1063 K. After an annealing time of 13 hours, the sample was slowly cooled with 10 K/h down to room temperature. A part of the white solid body retrieved from the synthesis run was ground in an agate mortar to a very fine powder that was used for powder X‐ray diffraction (PXRD) and thermal analysis. In the remaining part, single crystals of sufficient optical quality could be found. One of them was employed for the in‐situ high‐temperature diffraction data collections.

### 2.2 Powder X‐ray diffraction

PXRD data were acquired with a Bruker AXS D8‐Discover diffractometer in Bragg‐Brentano geometry using Cu‐K*α*_1_ radiation (40 kV, 40 mA) in the range between 5 and 135° 2θ with a counting time of 6 s per step and a step size of 0.01° 2θ. The device is equipped with a primary beam Ge(111) monochromator. An LYNXEYE silicon strip detector, as well as primary and secondary Soller slits, were used. The divergence slit was set to an opening angle of fixed 0.3°. The samples were prepared on a “background‐free” silicon single‐crystal sample holder with a circular sample cavity of 20 mm × 0.5 mm.

**FIGURE 1:**
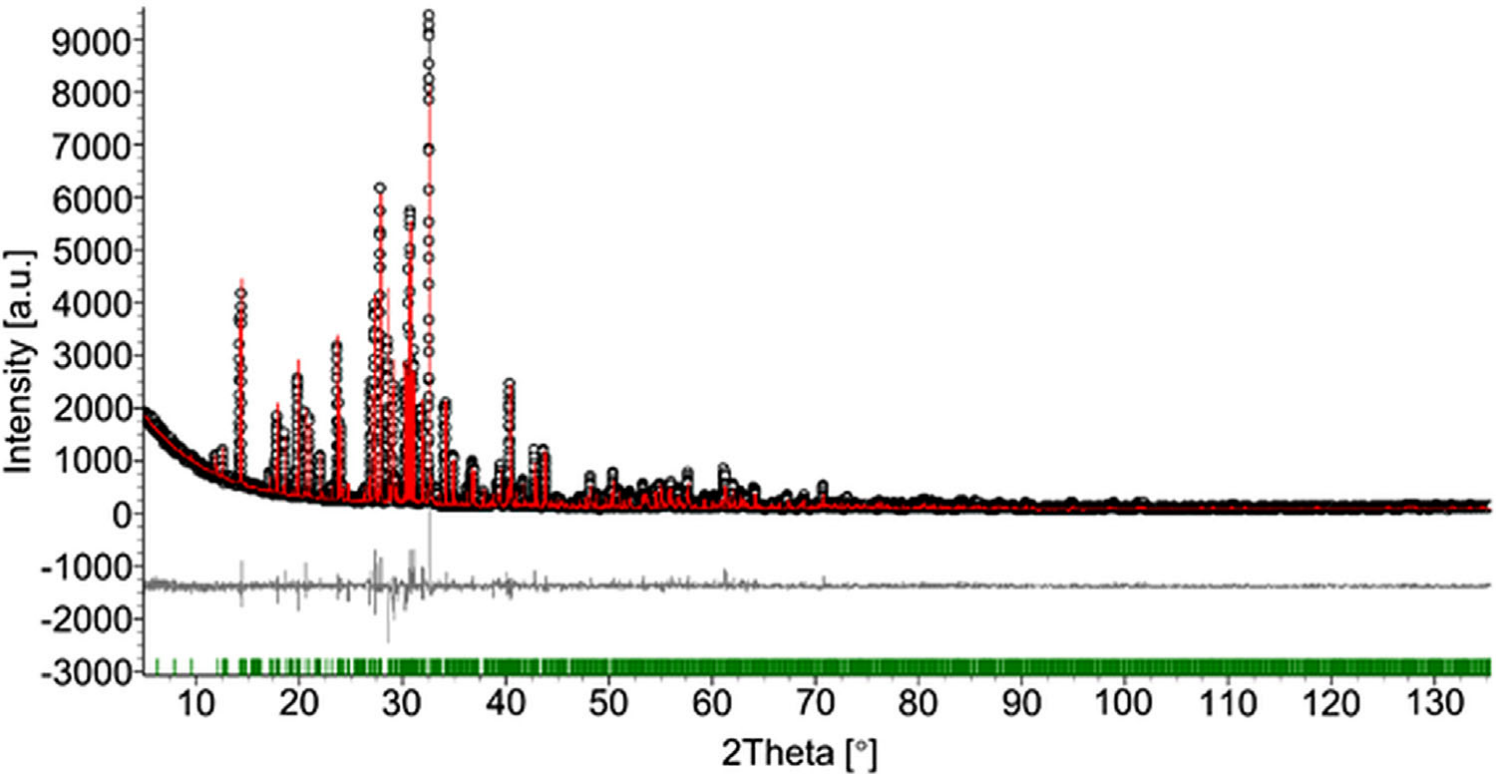
Rietveld fit of the powder diffraction pattern acquired for the sample prepared from the solid‐state reactions (Cu‐Kα_1_ radiation, Bragg‐Brentano θ−2θ geometry). Observed step intensities *y*_obs_ are represented by small blue circles. Calculated step intensities *y*_cal_: solid red line. The lower grey line shows the difference curve, *y*_obs—_ *y*_cal_. The green vertical bars below the diffraction pattern indicate the positions of the allowed reflections of K_4_CaSi_6_O_15_ of the ambient temperature modification.

Qualitative phase analysis was attempted with the 2018 release of the PDF‐4 Powder Diffraction File issued by the International Centre for Diffraction Data. Rietveld analysis was performed on the sample using the TOPAS Version 6.0 software to check the purity of the synthesized sample.^27^ The modeling of peak profiles was based on the fundamental parameter approach^28^. For a description of the background, Chebychev polynomials of the 9^th^ order were applied. The published crystal structure of K_4_CaSi_6_O_15_ at ambient temperature^22^ was used as the initial structure model for subsequent fitting. In the course of the refinement, lattice parameters and peak widths were optimized as well as atom occupancies on the split K‐positions. The March‐Dollase function was employed to address a preferred orientation parallel to (001). The refinement converged to a *R*‐values of 0.0842 for *R_p_* and 0.1243 for *R_wp_*. Figure 1 presents a visual comparison between the observed and calculated powder patterns. The very good agreement indicates that the sample has a very high purity and can be used as a starting material for heat capacity measurements.

### 2.3 Calorimetric methods

Low‐temperature heat capacities in the range of 2–300 K were measured with a commercially available relaxation calorimeter (the heat capacity option of the Quantum Design Physical properties measurement system—PPMS). The data were collected in triplicate at 60 different temperatures between 2 and 300 K, using a logarithmic spacing. The sample consisting of 14 mg was wrapped in a thin Al‐foil and compressed to a ∼0.5 mm thick pellet that was then attached to the sample platform of the calorimeter with Apiezon N‐grease, to facilitate the required thermal contact. Heat capacities around and above ambient T were collected using a Perkin Elmer Diamond differential scanning calorimeter (DSC). More details on both calorimetric techniques, as well as measuring and evaluation procedures, have already been described several times and will not be repeated here^29^, ^30^, ^31^, ^32^, ^33^. All calorimetric data are given in TablesS1 and S2.

### 2.4 In‐situ single‐crystal diffraction

In order to perform in‐situ high‐temperature diffraction studies, a crystal of excellent optical quality under a polarizing binocular was selected, cleaned in a droplet of water, and clamped in the narrowing part of a silica glass capillary with an inner diameter of 0.1 mm. Data collection was executed using a two‐circle Stoe IPDS‐II single‐crystal diffractometer, equipped with an image‐plate detector. For data collection at elevated temperatures, the crystal was heated by a furnace mounted on the ω axis providing a hot N_2_ gas flow. Good temperature stability was achieved by (i) a fixed gas stream outlet (located approximately 3 mm below the sample for all goniometer positions), (ii) a mass flow controller (providing a constant flow of 0.8 L/min N_2_), and (iii) a stabilized power supply unit. The program *htcontrol* was used to control the temperature of the diffraction experiments.^34^

All data collections were performed under the same conditions: the Mo tube was operated at 50 kV and 40 mA. A plane graphite monochromator and a 0.5 mm multiple pinhole collimator were utilized to direct the beam to the sample. The distance between the sample and the detector was set to 120 mm.

A first series of diffraction experiments was performed at 298, 373, 473, 573, 673, 773, 873, 973, and 1063 K. These data collections were performed in a 180° omega‐rotation, using 0.4° steps with 1.1 min of exposure time. As changes in the diffraction pattern were observed, which indicated the occurrence of two phase transitions, a second series of diffraction experiments was conducted, using smaller steps in temperature change and, in order to reduce the overall experimental time, faster data collections covering 70° in omega, using 1° steps with exposures of 2.5 minutes. Between 423 and 693 K, a total of 22 data collections were carried out, with temperature steps of 10 or 15 K.

The diffraction data acquired at 773 K were used to solve the crystal structure of the high‐*T* polymorph of K_4_CaSi_6_O_15_. Data reduction was performed using the X‐AREA software package,^35^ which included lattice‐parameter refinements, Lorentz, and polarization corrections. After integration, the intensities were subsequently corrected for absorption based on 39 indexed faces describing the morphology of the irregular fragment used for data collection.

Analysis of diffraction symmetry indicated monoclinic Laue group 2∕*m*. Since systematic absences pointed to the space group *P*2_1_/*c*, structure determination was successfully initiated in this symmetry using direct methods implemented in the program SIR2004.^36^ Subsequently, least‐squares refinements of the complete crystallochemically reasonable model were performed using the program SHELX‐97^37^ embedded in the WinGX program suite.^38^ The structure determination revealed that the crystalline material stable at 773 K indeed corresponds to a high‐temperature polymorph of K_4_CaSi_6_O_15_. In the course of the refinement, it became obvious, that one of the potassium cations in the asymmetric unit occupies a split position, which was considered assuming that the occupancies of both sites sum to 100 %. Furthermore, the anisotropic displacement parameters of the split atom pairs were restrained to be similar (SIMU command of the SHELX‐97 program). Final full‐matrix least‐squares refinement cycles including fractional coordinates as well as anisotropic displacement parameters for all atoms converged to a residual of *R*_1_ = 0.0368 for 131 parameters and 1435 reflections with *I* > 2*σ*(*I*) (Table 1). In Table 2, fractional atomic coordinates together with equivalent isotropic displacement factors and bond valence sums (BVS) of the cations are listed. Selected bond distances and bond angles, as well as anisotropic displacement factors, are given in Tables 3 and 4, respectively. Thermal ellipsoids of the atoms were well‐behaved showing expected orientations. For example, the largest principal axes of the prolate‐ or oblate‐shaped ellipsoids of the oxygen atoms were almost perpendicular to the corresponding Si‐O bonds.

**TABLE 1:** Crystal data and structure refinement for K_4_CaSi_6_O_15_ at 773 K.

Empirical formula	K_4_CaSi_6_O_15_	
Formula weight	605.02 g/mol	
Temperature	773(2) K	
Wavelength	0.71073 Å	
Crystal system	Monoclinic	
Space group	*P*2_1_/*c*	
Unit cell dimensions	*a* = 6.9469(4) Å	α = 90°
	*b* = 9.2340(5) Å	β = 93.639(4)°
	*c* = 12.2954(6) Å	γ = 90°
Volume	787.13(7) Å^3^	
*Z*	2	
Density (calculated)	2.553 g/cm^3^	
Absorption coefficient	1.991 mm^−1^	
*F*(000)	600	
Crystal form, size	Fragment, 0.35 × 0.30 × 0.20 mm3	
θ range for data collection	0.955−27.39°	
Index ranges	−8 < = h < = 8, −11 < = k < = 11, −14 < = l < = 14	
Reflections collected	5095	
Independent reflections (I > 2σ(I))	1435 [R(int) = 0.0379]	
Completeness to θ = 25.242°	99.3 %	
Refinement method	Full‐matrix least‐squares on F^2^	
Data / restraints / parameters	1435 / 6 / 131	
Goodness‐of‐fit on *F* ^2^	1.065	
Final *R* indices (*I* > 2*σ*(*I*))	*R*1 = 0.0368, w*R*2 = 0.0978	
*R* indices (all data)	*R*1 = 0.0392, w*R*2 = 0.1004	
Largest diff. peak and hole	0.481 and −0.350 e.Å^−3^	

**TABLE 2:** Atomic coordinates (×10^4^) and equivalent isotropic displacement parameters (Å^2^ × 10^3^) for K_4_CaSi_6_O_15_ at 773 K. *U*(eq) is defined as one third of the trace of the orthogonalized U_ij_ tensor. Bond valence sums (BVS) for the cations are given in valence units (v.u.). K(21) and K(22) correspond to partially occupied split positions (see text).

Atom	Wyckoff‐site	Occupancy	*x*	*y*	*z*	*U*(eq)	BVS
Ca(1)	2*d*	1.0	5000	0	5000	32(1)	1.94
K(1)	4*e*	1.0	3399(2)	−2974(1)	3136(1)	60(1)	0.80
K(21)	4*e*	0.63(3)	17(14)	−1780(20)	5250(4)	87(3)	0.86
K(22)	4*e*	0.37(3)	−550(30)	−2450(30)	5235(7)	82(4)	0.70
Si(1)	4*e*	1.0	−1569(1)	−312(1)	7528(1)	30(1)	4.32
Si(2)	4*e*	1.0	2171(1)	1384(1)	7311(1)	29(1)	4.37
Si(3)	4*e*	1.0	4376(1)	−3856(1)	5868(1)	28(1)	4.47
O(1)	4*e*	1.0	−1172(5)	−2024(3)	7759(3)	60(1)	
O(2)	4*e*	1.0	−2424(4)	31(3)	6365(2)	57(1)	
O(3)	4*e*	1.0	3636(5)	−2424(3)	5353(3)	65(1)	
O(4)	4*e*	1.0	574(4)	339(4)	7794(3)	72(1)	
O(5)	2*b*	1.0	5000	5000	5000	103(2)	
O(6)	4*e*	1.0	3823(4)	1412(4)	8276(3)	70(1)	
O(7)	4*e*	1.0	−2787(6)	313(5)	8473(3)	94(1)	
O(8)	4*e*	1.0	2880(5)	909(5)	6214(3)	83(1)	

**TABLE 3:** Bond lengths [Å] (with and without correction for thermal motion) as well as bond angles [°] in K_4_CaSi_6_O_15_ at 773 K. The distortion parameters for the tetrahedra and octahedra are also given (λ: Quadratic elongation; σ^2^: Angle variance).

Ca(1)‐O(8)	2.319(3)	Ca(1)‐O(8)	2.319(3)	Ca(1)‐O(2)	2.375(3)
Ca(1)‐O(2)	2.375(3)	Ca(1)‐O(3)	2.479(3)	Ca(1)‐O(3)	2.479(3)
<Ca(1)‐O>	2.391	λ	1.012	σ^2^	37.05
K(1)‐O(3)	2.768(4)	K(1)‐O(2)	2.876(3)	K(1)‐O(4)	2.948(4)
K(1)‐O(6)	3.040(4)	K(1)‐O(5)	3.1093(10)	K(1)‐O(7)	3.166(5)
K(1)‐O(1)	3.180(3)	K(1)‐O(6)	3.192(4)	K(1)‐O(8)	3.270(5)
K(21)‐O(3)	2.578(7)	K(21)‐O(8)	2.734(5)	K(21)‐O(2)	2.802(6)
K(21)‐O(2)	3.127(18)	K(21)‐O(1)	3.252(9)	K(21)‐O(1)	3.309(7)
K(21)‐O(8)	3.352(19)				
K(22)‐O(8)	2.733(7)	K(22)‐O(3)	2.90(2)	K(22)‐O(2)	3.020(15)
K(22)‐O(1)	3.086(13)	K(22)‐O(4)	3.168(14)	K(22)‐O(1)	3.186(9)
K(22)‐O(6)	3.19(2)	K(22)‐O(7)	3.417(8)		
Si(1)‐O(2)	1.546(3)	Si(1)‐O(7)	1.589(3)	Si(1)‐O(4)	1.619(3)
Si(1)‐O(1)	1.627(3)				
<Si(1)‐O>	1.595	λ	1.008	σ^2^	35.31
Si(2)‐O(8)	1.529(3)	Si(2)‐O(6)	1.598(3)	Si(2)‐O(4)	1.612(3)
Si(2)‐O(1)	1.625(3)				
<Si(2)‐O>	1.591	λ	1.006	σ^2^	25.17
Si(3)‐O(3)	1.540(3)	Si(3)‐O(5)	1.5815(8)	Si(3)‐O(6)	1.602(3)
Si(3)‐O(7)	1.605(3)				
<Si(3)‐O>	1.582	λ	1.003	σ^2^	14.56
Ca‐O and Si‐O bond lengths corrected for the thermal motion based on a simple rigid bond correction					
Ca(1)‐O(8)	2.352	Ca(1)‐O(8)	2.352	Ca(1)‐O(2)	2.391
Ca(1)‐O(2)	2.391	Ca(1)‐O(3)	2.499	Ca(1)‐O(3)	2.499
Si(1)‐O(2)	1.572	Si(1)‐O(7)	1.648	Si(1)‐O(4)	1.657
Si(1)‐O(1)	1.654				
Si(2)‐O(8)	1.581	Si(2)‐O(6)	1.636	Si(2)‐O(4)	1.652
Si(2)‐O(1)	1.653				
Si(3)‐O(3)	1.576	Si(3)‐O(5)	1.651	Si(3)‐O(6)	1.641
Si(3)‐O(7)	1.666				
O‐Si‐O angles					
O(2)‐Si(1)‐O(7)	114.25(19)	O(2)‐Si(1)‐O(4)	113.91(18)	O(7)‐Si(1)‐O(4)	104.1(2)
O(2)‐Si(1)‐O(1)	114.30(17)	O(7)‐Si(1)‐O(1)	108.5(2)	O(4)‐Si(1)‐O(1)	100.49(17)
O(8)‐Si(2)‐O(6)	113.8(2)	O(8)‐Si(2)‐O(4)	114.9(2)	O(6)‐Si(2)‐O(4)	102.41(18)
O(8)‐Si(2)‐O(1)	111.9(2)	O(6)‐Si(2)‐O(1)	107.98(18)	O(4)‐Si(2)‐O(1)	105.03(19)
O(3)‐Si(3)‐O(5)	113.27(15)	O(3)‐Si(3)‐O(6)	111.33(19)	O(5)‐Si(3)‐O(6)	108.10(14)
O(3)‐Si(3)‐O(7)	113.3(2)	O(5)‐Si(3)‐O(7)	104.36(18)	O(6)‐Si(3)‐O(7)	106.0(2)
Si‐O‐Si angles					
Si(2)‐O(1)‐Si(1)	143.4(2)	Si(2)‐O(4)‐Si(1)	142.5(2)	Si(3)‐O(5)‐Si(3)	180.00
Si(2)‐O(6)‐Si(3)	168.4(3)	Si(1)‐O(7)‐Si(3)	163.3(3)		
O‐Ca‐O angles					
O(8)‐Ca(1)‐O(8)	180	O(8)‐Ca(1)‐O(2)	91.23(12)	O(8)‐Ca(1)‐O(2)	88.77(12)
O(8)‐Ca(1)‐O(2)	88.77(12)	O(8)‐Ca(1)‐O(2)	91.23(12)	O(2)‐Ca(1)‐O(2)	180
O(8)‐Ca(1)‐O(3)	87.07(15)	O(8)‐Ca(1)‐O(3)	92.93(15)	O(2)‐Ca(1)‐O(3)	99.58(10)
O(2)‐Ca(1)‐O(3)	80.42(10)	O(8)‐Ca(1)‐O(3)	92.93(15)	O(8)‐Ca(1)‐O(3)	87.07(15)
O(2)‐Ca(1)‐O(3)	80.42(10)	O(2)‐Ca(1)‐O(3)	99.58(10)	O(3)‐Ca(1)‐O(3)	180

**TABLE 4:** Anisotropic displacement parameters (Å^2^ × 10^3^) for K_4_CaSi_6_O_15_ at 773 K. The anisotropic displacement factor exponent takes the form: −2π^2^[ *h* ^2^ a*^2^ *U*_11_ + … + 2 h k a* b* *U*_12_].

	*U*_11_	*U*_22_	*U*_33_	*U*_23_	*U*_13_	*U*_12_
Ca(1)	31(1)	37(1)	28(1)	‐3(1)	‐2(1)	4(1)
K(1)	70(1)	46(1)	63(1)	12(1)	‐1(1)	‐4(1)
K(21)	55(2)	143(6)	63(1)	‐7(2)	‐1(1)	34(3)
K(22)	66(5)	127(8)	51(2)	16(3)	1(2)	29(5)
Si(1)	28(1)	27(1)	33(1)	0(1)	‐2(1)	1(1)
Si(2)	27(1)	28(1)	31(1)	‐1(1)	‐3(1)	1(1)
Si(3)	30(1)	29(1)	24(1)	‐1(1)	‐1(1)	1(1)
O(1)	67(2)	30(1)	80(2)	7(1)	‐19(2)	‐7(1)
O(2)	61(2)	64(2)	44(2)	5(1)	‐20(1)	8(1)
O(3)	67(2)	42(2)	83(2)	16(2)	‐22(2)	4(1)
O(4)	51(2)	74(2)	86(2)	32(2)	‐20(2)	‐31(2)
O(5)	135(5)	87(4)	89(4)	‐54(3)	22(4)	20(4)
O(6)	49(2)	88(2)	68(2)	0(2)	‐32(2)	0(2)
O(7)	85(3)	134(4)	63(2)	‐15(2)	18(2)	56(3)
O(8)	84(2)	117(3)	50(2)	‐17(2)	14(2)	46(2)

To include the impact of temperature on the bond distances involving the cations residing in the barycenters of the comparatively rigid tetrahedra and octahedra, the values of the Si‐O and Ca‐O bond lengths have been corrected for thermal motion using the so‐called “simple rigid bond” model proposed by Downs et al.^39^ (see Table 3). For calculating bond valence sums, the program VaList^40^ was applied in combination with the parameter sets of Brown and Altermatt^41^ for Ca–O and K–O, as well as Brese and O'Keeffe^42^ for Si–O bonds. Figures showing the crystal structure have been prepared using the program VESTA 3.^43^

## 3 RESULTS

### 3.1 Heat capacity and thermodynamic properties of K_4_CaSi_6_O_15_

The standard third‐law entropy at 298.15 K, *S*°, of K_4_CaSi_6_O_15_ was calculated from the experimentally determined low‐temperature *C*_p_‘s by numerically solving the integral (1) assuming that *S^T^* ^= 0K^ is 0.

(1)

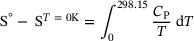




**FIGURE 2:**
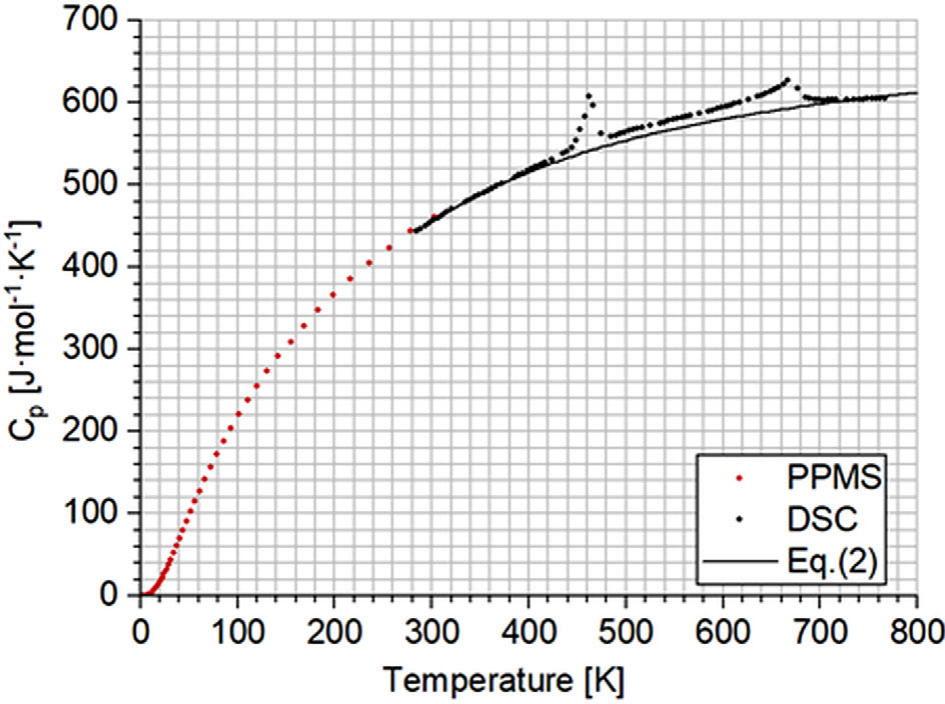
Heat capacity of K_4_CaSi_6_O_15_ as a function of temperature between 2 and 800 K.

By performing the integration between 2 K and 298.15 K, the resulting *S* ^o^ is 524.3 ± 3.7 J·mol^−1^·K^−1^. As *C*_p_ below 2 K has very low absolute values < 0.02 J·mol^−1^·K^−1^, the entropy increment from 0 to 2 K could be neglected. Standard entropy determinations based on the present PPMS‐measured *C*_p_ data are expected to be accurate within 0.7%,^32^ the uncertainty of *S* ^o^ is ca. 3.7 J·mol^−1^·K^−1^. A graphical representation of the data is shown in Figure 2. The agreement between the PPMS and DSC data around room temperature is good, the deviations are < 0.7%.

**FIGURE 3:**
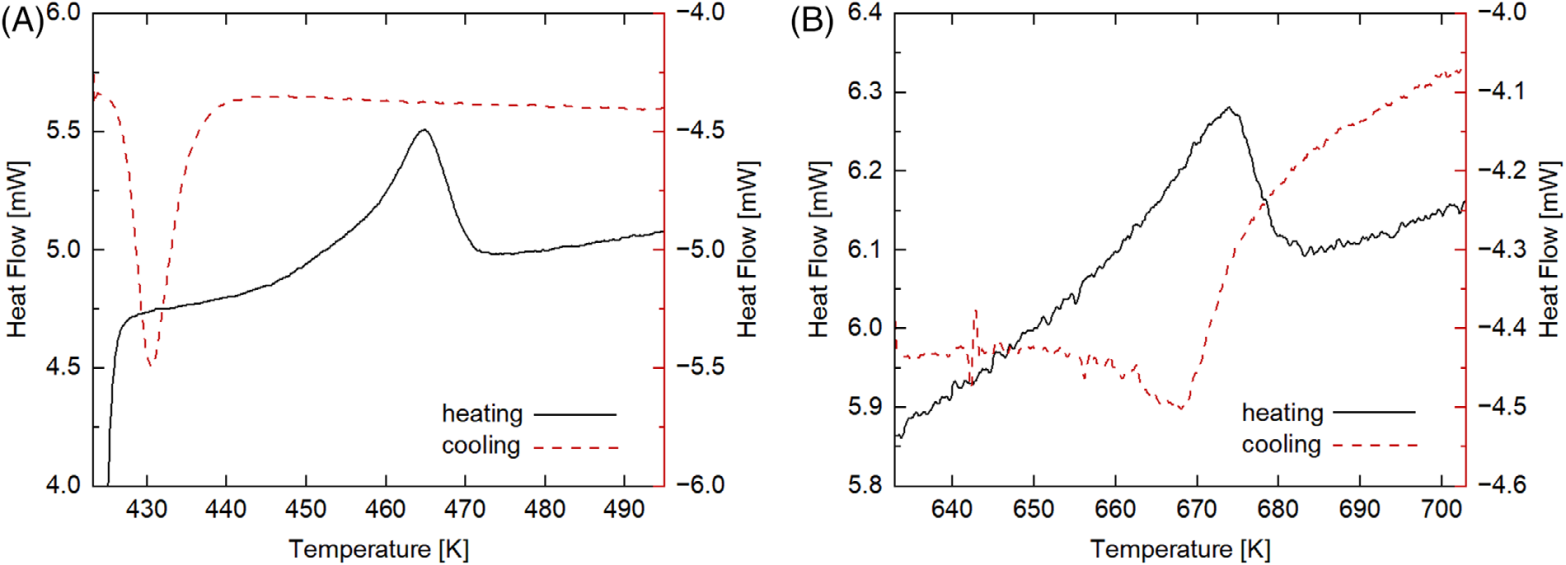
Differential scanning calorimetry data for the heating and cooling cycle in the temperature regions around the phase transitions. The temperature hysteresis for the 1^st^ transition is ca. 34 K (A) and ca. 4 K for the 2^nd^ transition (B). The heating and cooling rates were 10 K/min.

Two *C*_p_ anomalies are clearly seen at about *T*_1_ = 462 K and *T*_2_ = 667 K in the DSC‐measured heat capacities (Figure 2), indicating two structural phase transitions occurring in the K_4_CaSi_6_O_15_ compound above ambient *T*. For the one at *T*_1_, the hysteresis is 33 K, while for the latter it is approximately 5 K (Figure 3). In order to represent the heat capacity of the high‐*T* polymorph in the *P*2_1_/*c* structure (see below), we have fitted the lower‐ and uppermost DSC data, which are most likely not affected by the two‐phase transitions and thus should represent lattice *C*_p_, to a Berman & Brown‐type *C*_p_‐polynomial^44^, yielding (in J·mol^−1^·K^−1^):

(2) $$\begin{array}{ccc} C_{p} & = & 735.3-2754.85T^{-0.5}-1.99063\cdot 10^{7}T^{-2}\\ & & +\;2.71429\cdot 10^{9}T^{-3} \end{array}$$

**FIGURE 4:**
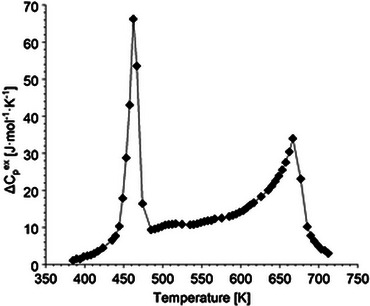
The excess‐C_p_ (ΔC_p_^ex^) as a function of temperature between 385 and 727K. ΔC_p_^ex^ is calculated by subtracting the base‐line C_p_ (C_p_^baseline^) obtained from Equation (2) from the calorimetric data acquired using differential scanning calorimetry (C_p_^DSC^), i.e., ΔC_p_^ex^ = C_p_^DSC^‐C_p_^baseline^.

This polynomial (Figure 2) served to compute a baseline *C*_p_ in order to model the heat capacity of the two structural phase transitions. Above 667 K, equation (2) can be used to calculate the *C*_p_ of the high‐*T* phase up to high temperatures > 1000 K. The resulting excess‐*C*_p_ (i.e., Δ*C*_p_^ex^ = *C*_p_^DSC^‐*C*_p_^baseline^) in the temperature region of the two‐phase transitions is shown in Figure 4. Taking a temperature of 485 K as a switchover between the two transitions, numerical integration of Δ*C*_p_^ex^ and Δ*C_p_* ^ex^/*T* over the temperature intervals 385 to 485 K and 485 to 727 K yielded transition enthalpy (Δ*H* ^tr^) and entropy (Δ*S* ^tr^) values. For the 1^st^ transition, Δ*H* ^tr1^ = 1.48 kJ·mol^−1^ and Δ*S* ^tr1^ = 3.25 J·mol^−1^·K^−1^, whereas Δ*H* ^tr2^ = 3.33 kJ·mol^−1^ and Δ*S* ^tr2^ = 5.23 J·mol^−1^·K^−1^ was determined for the 2^nd^ transition. For thermodynamic calculations in the stability field of the high‐*T P*2_1_/*c‐*phase, these transition properties (in sum Δ*H* ^tr^ = 4.81 kJ·mol^−1^ and Δ*S* ^tr^ = 8.48 J·mol^−1^·K^−1^) have to be added to the enthalpy and entropy function of K_4_CaSi_6_O_15_, whose temperature dependencies can be computed using equation (2).

### 3.2 Crystal structure for K_4_CaSi_6_O_15_ at 773 K

In the high‐temperature form of K_4_CaSi_6_O_15_, three crystallographically independent Si sites can be distinguished. They bond with their oxygen ligands to form [SiO_4_]‐tetrahedra, building a three‐dimensional network by sharing common corners. The framework, however, is interrupted because each tetrahedron has only three bridging oxygen atoms (Obr) connected to the other tetrahedra, that is, it contains only the so‐called tertiary tetrahedral units (Q^3^). A Si:O ratio of 1:2.5 is rare in framework silicates, but well known in many single‐layer, double‐chain, and a few double‐ring silicates.^45^ Notably, the connectivity of the [SiO_4_]‐groups in the sub‐structure of the silicate tetrahedra is identical to the one observed in ambient temperature K_4_CaSi_6_O_15_.^22^

**FIGURE 5:**
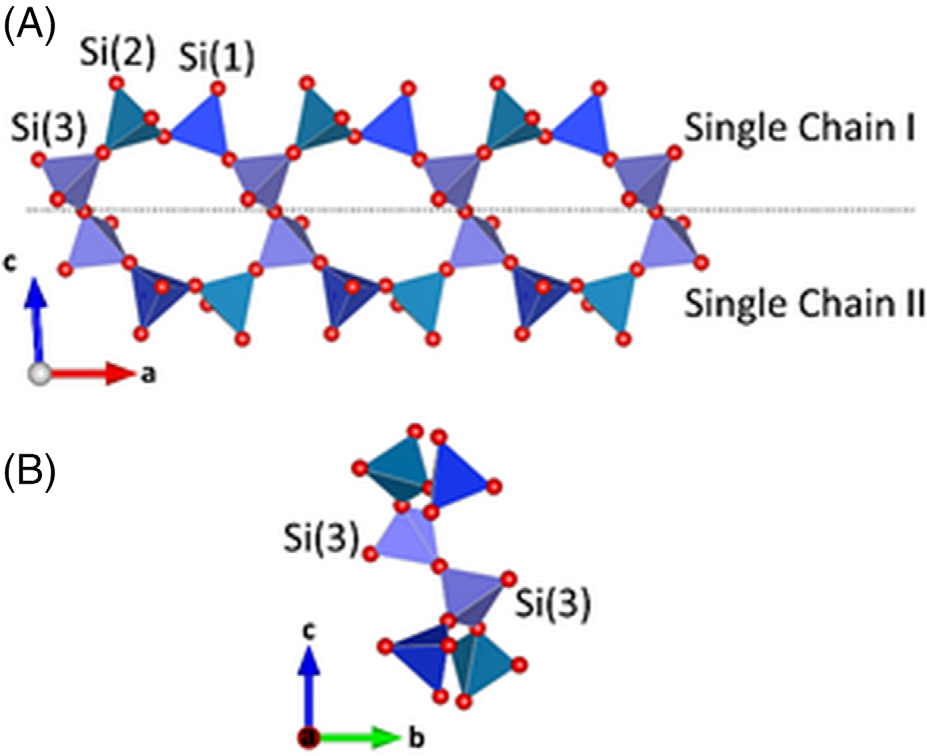
Projections of two of the fundamental *dreier* single chains belonging to the tetrahedral network (A) perpendicular and (B) parallel to the chain direction. Oxygen atoms are given as red spheres. The symmetrically independent tetrahedra are given in blue, cyan, and purple.

**FIGURE 6:**
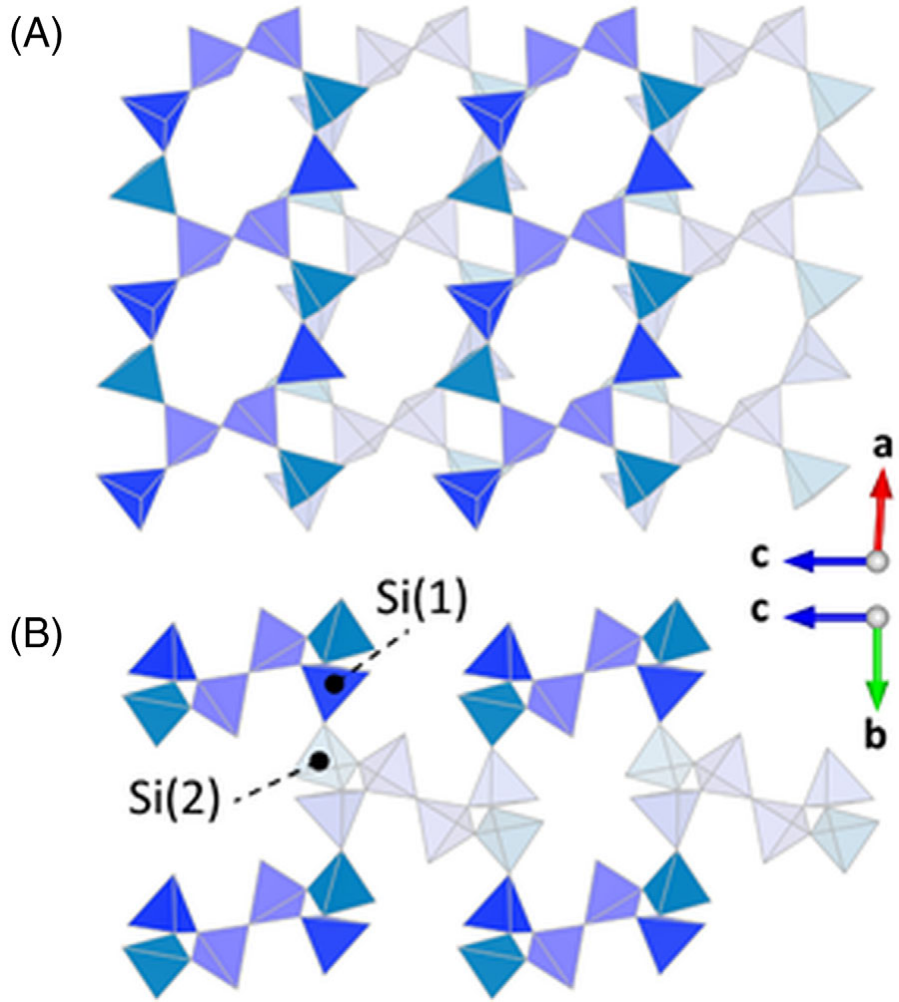
Sequence of three adjacent layers parallel (010) containing eight‐membered rings (opaque tetrahedra: y≈0; transparent tetrahedra: y≈1/2).

The basic building blocks of the present framework are *dreier* single chains running parallel to [100], with a translational period of about 6.94 Å, corresponding to the lattice parameter *a*. Two equivalent tetrahedra (Si(3)) sharing common oxygen corners located on a center of inversion join two adjacent single chains forming puckered eight‐membered rings (see Figure 5A,B). The condensation of these rings results in the formation of layers parallel to (010) throughout three‐dimensional space. The layers are located at *y* ≈ 0 and *y* ≈ ½, respectively. The connection between adjacent layers via two symmetrically independent tetrahedra (around Si(1) and Si(2)) with a common oxygen corner leads to an ABABAB‐type stacking sequence and eventually to the formation of the entire framework (see Figure 6A,B). Applying the aforementioned simple rigid bond correction for thermal motion, the Si‐O distances within each tetrahedron conform to the expected crystallographic trends for Q^3^ moieties, that is, the Si‐Obr bond lengths (1.636–1.666 Å) are all relatively longer than the distances (1.572–1.581 Å) between Si and the non‐bridging oxygen atoms. This type of shortening is due to the well‐known stronger attraction between O and Si compared to O and K/Ca in the structure. The average O‐Si‐O angles of the three tetrahedra are very close to the ideal value of 109.47°, while the individual O‐Si‐O angles range from 100° to 115°, indicating that the tetrahedra deviate considerably from regularity. The spread is similar to the values reported in the literature for other interrupted silicate frameworks. For a quantitative assessment of the polyhedral distortions the quadratic elongation λ and the angle variance σ^2^ can be used.^46^ For the three independent tetrahedra, the corresponding values of these parameters are λ = 1.008 and σ^2^ = 35.31 for Si(1), λ = 1.006 and σ^2^ = 25.17 for Si(2), λ = 1.003 and σ^2^ = 14.56 for Si(3), which are comparable with those obtained in the room temperature phase. The Si–O–Si angles exhibit a spread between 142.5° and 180° with an average value of 159.5°. The bond valence sums (BVS) of the silicon atoms indicate an overbonding, that is, their BVS is higher than the expected value of 4 valence units, while the potassium sites exhibit an underbonding (see Table 2). The BVS value for the calcium cation compares well with its valence. However, one has to keep in mind that the bond valence parameters that have been used for the calculations (see the Introduction) refer to values that have been determined at ambient temperature.

Charge compensation is achieved by incorporating potassium and calcium cations. They are distributed among three crystallographically independent sites that are coordinated by six to nine oxygen ligands (see Table 3). The coordination polyhedra around the Ca‐ions represent distorted octahedra (λ = 1.012 and σ^2^ = 37.05), while the oxygen environments of the potassium cations are more irregular and involve between seven and nine next anions.

A slightly different understanding of the structure can be obtained when the [CaO_6_]‐octahedra are included in the construction of a more complex network. In this new mixed tetrahedral‐octahedral (MTO) framework, every octahedron shares six corners with adjacent tetrahedra. A detailed topological analysis of the MTO‐net, including coordination sequences and extended point symbols, has been performed with the help of the program ToposPro.^47^ Therefore, the framework consisting of the [SiO_4_]‐ and [CaO_6_]‐units is described by a graph composed of the vertices (Ca‐, Si‐ and O‐atoms) as well as edges (bonds) between them. The nodes of the graph can be classified according to their coordination sequences {N*_k_*}.^48^ They represent a set of integers {N*_k_*} (*k* = 1,…, m), where N*_k_* is the number of sites in the *k*‐th coordination sphere of the M/T‐ or O‐atom that has been selected to be the central one. The corresponding values for the crystallographically independent Ca‐ and Si‐sites up to m = 10 (without the oxygen nodes) are summarized in Table 5. Furthermore, the extended point symbols^49^ listing all shortest circuits for each angle for any non‐equivalent atom have been determined. The results are also given in Table 5.

**TABLE 5:** Coordination sequences {N*_k_*} of the Si‐ and Ca‐nodes (without oxygen atoms) of the MTO‐framework in K_4_CaSi_6_O_15_ as well as the extended point symbols.

Si/Ca‐ sites	Coordination sequences {N*_k_*} (*k* = 1–10)										Extended point symbol
	*1*	*2*	*3*	*4*	*5*	*6*	*7*	*8*	*9*	*10*	
Si(1)	4	12	29	48	75	113	147	193	248	301	4.6.4.6.5.6
Si(2)	4	12	27	47	75	110	147	194	244	303	4.6.4.6_2_.5.7
Si(3)	4	12	27	48	76	105	147	192	246	300	4.5.4.5.6_2_.7_5_
Ca(1)	6	14	26	54	78	106	152	198	240	308	4.4.4.4.5.5.6.6.6_2_.6_2_.7_4_.7_4_.8_2_.8_2_.9_10_

**FIGURE 7:**
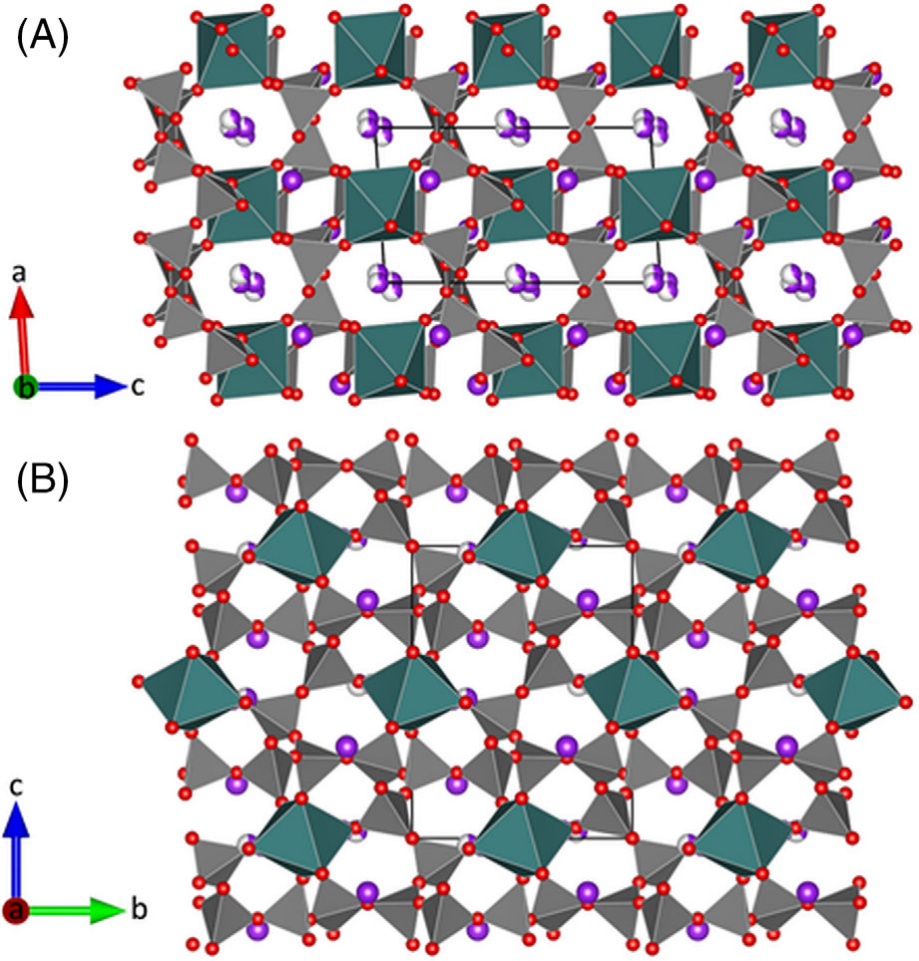
Projections of the whole crystal structure of K_4_CaSi_6_O_15_ parallel to (A) [010] and (B) [100], respectively. [CaO_6_]‐octahedra are shown in green. Purple spheres represent the potassium cations. White sectors within the spheres indicate partial occupancy of the split positions.

The potassium cations in turn are located in the voids of the MTO‐net. A careful analysis of the site occupancies and residual electron densities during the refinements revealed that the K2‐position is split into two sites (K(21), K(22)) with K–K distances of approximately 0.732 Å. They are concentrated at about *x* ≈ 0 and *z* ≈ 1/4 within tunnels formed by the eight‐membered tetrahedral rings running parallel to [010]. On the other hand, the fully occupied K‐sites are located in slabs parallel to (100) at *x* ≈ 1/3 (see Figure 7A,B). As mentioned above, the bond valence sums suggest an underbonding of the K‐positions, that is, they are too small and do not fit very well into the voids between the surrounding [SiO_4_]‐ and [CaO_6_]‐units of the MTO framework.

### 3.3 Phase transitions of K_4_CaSi_6_O_15_

**FIGURE 8:**
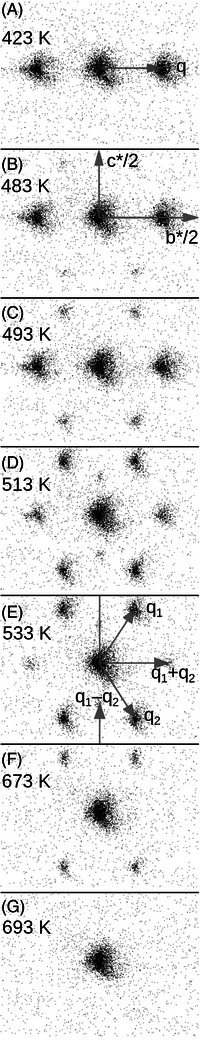
Diffraction patterns of K_4_CaSi_6_O_15_ at elevated temperatures mapped into a single cell (−0.5 < h,k,l < 0.5), representing the frequency distributions of all diffraction peaks that have been recorded. Additional reflections were interpreted as q, q_1_, q_2_, q_1_ + q_2_, and q_1_ – q_2_ (see text).

A peak search was performed for all frames of each measurement belonging to the 423–693 K HT data collection series, using the same search parameters.^35^ The basic unit‐cell (*a* = 6.9, *b* = 9.1, *c* = 12.2 Å and β = 93.7°) observed for the HT form was employed for assigning *hkl* indices to the peaks that were detected. To account for deviations from the exact nodes of the corresponding reciprocal lattice, floating‐point numbers are used for the allocated indices. Plotting the resulting peak lists by (i) neglecting the integer part and (ii) mapping the indices into a range of −0.5 < *h*,*k*,*l* < 0.5 results in a very concise three‐dimensional representation of the frequency distributions of all diffraction peaks that have been recorded and allows for convenient analysis of those peaks that cannot be explained with the basic unit‐cell. Figure 8A–G show projections of the 3‐dimensional distributions along a*. The central cluster in each figure represents the positions of the lattice nodes belonging to the basic unit cell. In Figure 8A (ambient temperature) two more clusters can be seen, at positions ±⅓·b*. From 453 K on, four additional clusters occur, whose densities increase (Figure 8B,C) with increasing temperature, while the clusters at ±⅓·b* become less densely populated with peaks (Figure 8C–E). At about 503 K two new clusters occur (Figure 8D,E). Starting from 548 K, the satellite reflections become weaker, and the corresponding clusters are thinning out until they have disappeared at 673 K (Figure 8F,G).

The interpretation of the aforementioned phenomena in reciprocal space supports the existence of two‐phase transformations in K_4_CaSi_6_O_15_. The unit cell of the HT form represents the common basic or average cell of all three phases. Under ambient conditions, a 3 × *b* superstructure is observed,^22^ that is, additional superstructure reflections occur which can be indexed with a q‐vector of (0, ⅓, 0), as can be seen in Figure 8A. At 533 K (Figure 8E), the diffraction pattern is more complex and can be explained by the existence of a (3+2)‐dimensional incommensurately modulated structure, using two q‐vectors q_1_ = (α, β, γ) and q_2_ = (−α, β, −γ), with α = 0.057, β = 0.172 and γ = 0.379. The two “weaker” pairs of peak clusters in Figure 8E can be explained with the combinations q_1_+q_2_ and q_1_‐q_2_ as well as the corresponding opposite directions. According to the diffraction pattern and the observed extinction conditions, the (3+2)‐dimensional superspace group *P*2_1_/*c*(α,β,γ)00(‐α,β,−γ)00 [No. 14.2.16.6]^50^ seems a match. Preliminary attempts to refine the modulated structure are promising. However, a detailed investigation of the (3+2)‐dimensional modulated phase will be the topic of another study.

In an intermediate temperature range (453‐523 K), the ambient temperature polymorph and the intermediate (3+2)‐dimensional phase seems to co‐exist. The first‐order satellites (superstructure reflections of the ambient temperature phase) defined by q almost perfectly overlap with the q_1_+q_2_ satellites of the (3+2)‐dimensional phase. This can be seen in Figure 8B–D in the changes in the peak density of the corresponding clusters. The observed onset and temperature range of the satellite reflections is in close agreement with the two effects observed in the thermal analysis (approximately 462 and 667 K).

Lattice parameter refinements—based on the basic unit cell—were carried out for all 22 data collections of the second series of HT diffraction experiments in the range of 423–693 K (see Figure S1). The evolution of the unit‐cell volume increases smoothly and almost linearly with temperature. Below 483 K and above 523 K, the *a* lattice parameter exhibits an increasing trend. However, between 483 and 523 K, a distinct step involving a decrease is observed. Fitting the data to linear functions, the rate of increase changes from 3.3(2) x 10^−5^ [Å/K] (for *T* < 483 K) to 6.9(2) x 10^−5^ [Å/K] (for *T* > 523 K). For the *b*‐direction, a discontinuity can be observed in the same temperature region. Actually, the rate of increase decreases from about 2.74(5) x 10^−4^ [Å/K] (for *T* < 483 K) to 2.08(5) x 10^−4^ [Å/K] (for *T* > 523 K). On the other hand, the *c* unit‐cell parameter exhibits a noticeable plateau in the abovementioned critical temperature range between 483 and 523 K. Finally, the monoclinic angle *β* decreases with increasing temperature, showing no significant discontinuities.

## 4 DISCUSSION

For crystalline materials undergoing temperature‐induced phase transitions, the high‐temperature form usually adapts a structure with a higher symmetry when compared with its low‐temperature counterpart. This rule also holds for the present compound. Neglecting the complex intermediate modulated phase, direct comparison between the high‐ and ambient temperature modifications of K_4_CaSi_6_O_15_ reveals, that the reduction in symmetry from *P*2_1_/c (point group 2/*m, a* = 6.9469(4) Å*, b* = 9.2340(5) Å, *c* = 12.2954(6) Å, β = 93.639(4)°) to *Pc* (point group *m, a* = 6.9299(2) Å*, b* = 27.3496(9) Å, *c* = 12.2187(5) Å, β = 93.744(3)°)^22^ involves two components: (i) loss of point group symmetry by a factor two and (ii) loss of translation symmetry by a factor three, that is, tripling of the *b* lattice parameter.

In summary, the high‐temperature and room‐temperature polymorphs are in a group‐subgroup relationship and the *Pc* structure represents a general subgroup index of 6. The connection between the basis vectors of the crystal structures belonging to the space groups *G* = *P*2_1_/*c* and *H* = *Pc* (subgroup) can be described by the following equations: **a** *_H_* = **a** *_G_*; **b** *_H_* = 3·**b** *_G_*; **c** *_H_* = **c** *_G_*.

For a more detailed analysis of the distortion relating the two structures, the program STRUCTURE RELATIONS accessible via the Bilbao Crystallographic Server has been employed^51^. For this purpose, it is necessary to describe the high‐symmetry structure in the most similar configuration to the low‐symmetry structure. For the present case, additional complexity with respect to finding the appropriate origin within the **a**—**c** plane is due to the polar character of *Pc*. Actually, the final calculated origin shift was (0.4604, ‐¼, 0.0089). In summary, one can say that the atomic coordinates of the high‐ and the ambient temperature structures of K_4_CaSi_6_O_15_ are related by the following 4 × 4 transformation matrix:

$$\left(\begin{array}{cc} \begin{array}{cc} 1 & \;0\\ 0 & \;3 \end{array} & \;\begin{array}{cc} 0 & \;0.4604\\ 0 & \;-0.25 \end{array}\\ \begin{array}{cc} 0 & \;0\\ 0 & \;0 \end{array} & \;\begin{array}{cc} 1 & \;0.0089\\ 0 & \;1 \end{array} \end{array}\right)$$

**FIGURE 9:**
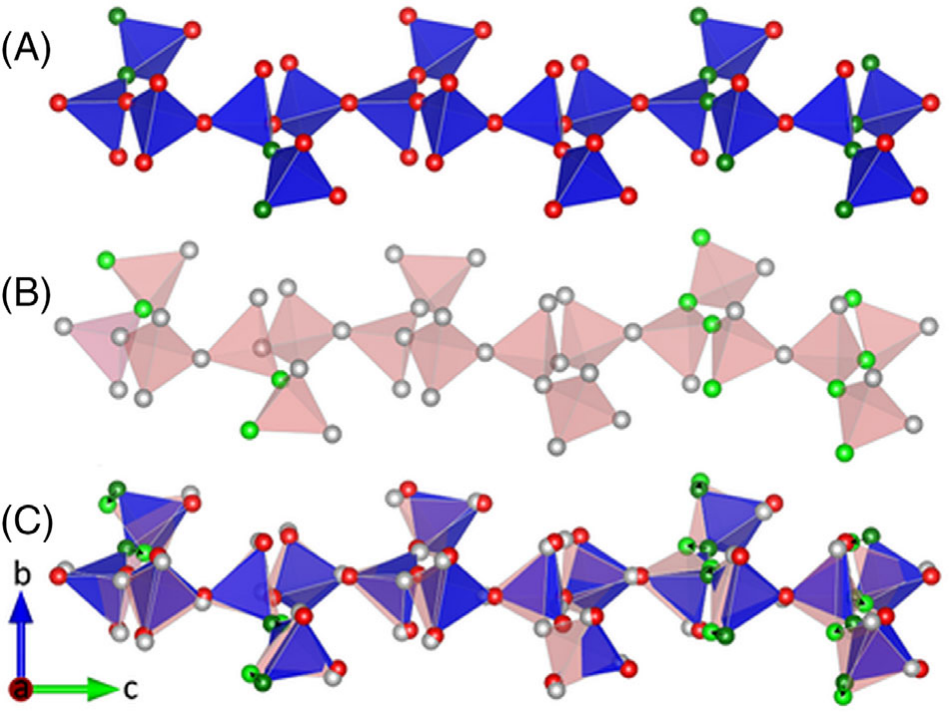
Projections of the tetrahedral chains along [100]: (A) Three unit‐cells in the high‐temperature structure; (B) a single unit‐cell in the room temperature structure; (C) overlay of the high temperature and room temperature structures. All tetrahedra are represented by translucent pink color for the structure at room temperature. The different colored spheres represent oxygen atoms, among which the ones with the top 10 displacement lengths are highlighted in dark green and green, respectively. The black arrows represent the projections of the displacement vectors in the plane parallel to (100).

In the next step of the analysis, pairs of atoms were identified in both structures, which are mapped onto each other using the above‐mentioned transformation matrix. The shift vectors between the paired atoms and their magnitudes can be used to explain the degree of distortion in the bond angles and bond lengths during the displacive phase transition. Table S3 lists the corresponding values of the atomic displacements. Taking into account the calcium, silicon, and potassium atoms, the shifts range from 0.031 to 0.374 Å with an average displacement of 0.217, 0.210, and 0.228 Å, respectively. In comparison, the oxygen atoms show relatively larger distortions. Some of them are particularly pronounced, with 10 oxygen atoms showing displacements that exceed 0.5 Å being considered the main contributors to the displacive transition (see Figure 9). A striking distortion among the anions refers to the oxygen O(5) located in the center of inversion of space group *P* 2_1_/*c*. The occupation of this special Wyckoff position implies the presence of a straight Si‐O(5)‐Si bond angle. Notably, the disc‐shaped thermal ellipsoid of O(5) exhibits the strongest anisotropy of all anions, which may point to the existence of either static or dynamic disorder. If so, the Si‐O(5)‐Si angle of 180° would represent an average value. According to Liebau,^45^ linear Si‐O‐Si linkages are energetically unfavorable. In the ambient‐temperature polymorph, the corresponding three bond angles involving the oxygens O(12), O(36), and O(39) have clearly tilted away from 180° to produce nonlinear arrangements with values of 149°, 146°, and 153°, respectively. The global amount of distortion can be expressed using the degree of lattice distortion parameter *S* = 0.0049 and the average displacement of all corresponding atom pairs in both structures which has a value of 0.293 Å.

Finally, the displacement field present in the low‐symmetry structure was decomposed into contributions from different modes, whose symmetries are given by the irreducible representations (irreps) of the space group of the parent or high‐symmetry phase. Using the AMPLIMODES^52^, ^53^ program, a total of four irreps were identified that are involved in the transition: Λ_1_, Λ_2,_ $\Gamma_{1}^{+}$and $\Gamma_{2}^{-}$, respectively. Actually, $\Gamma_{1}^{+}$ is the trivial fully symmetrical distortion that retains the symmetry of the high‐symmetry phase. The primary mode of the transition corresponds to the irrep Λ_1_ of space group *P*2_1_/*c* associated with the point Λ (0,⅓,0) inside the first Brillouin zone. The absolute amplitude (normalized with respect to the primitive unit cell of the high‐symmetry structure) of this component of the global distortion is 2.222 Å. The relevant values of the other modes are as follows: 0.570 (for $\Gamma_{1}^{+}$), 0.531 (for $\Gamma_{2}^{-}$) and 0.628 (for Λ_2_). When comparing the values of the magnitudes, the Λ_1_‐mode is about 3.5 to 4.2 times larger than the other modes, indicating that the onset of this mode triggers the transition. However, for a complete understanding, the contributions of the remaining three modes have to be considered as well. In summary, one can say, that the displacive phase transition is induced by an interplay between the partially occupied potassium sites within the channel‐like cavities and the surrounding MTO framework.

The abovementioned described discontinuities in the lattice parameters *a* and *b* are definitely related to the structural relaxations within the ***a***–***b***‐plane during the transformation to the 3+2‐dimensionally modulated phase.

## 5 CONCLUSIONS

With regard to K_4_CaSi_6_O_15_, revealing the presence of two structural transitions and their specific structures at elevated temperatures is of vital importance for a better comprehension of the phase behavior within the K_2_O‐CaO‐SiO_2_ ternary system. Laboratory molar low‐ and high‐temperature heat capacities were measured and the standard third‐law entropy of K_4_CaSi_6_O_15_ was determined, as well as the transition enthalpy and entropy properties that result from the heat‐capacity anomalies associated with the two‐phase transitions occurring at 462 K and at 667 K. For the computation of *C*_p_ above 298.15 K, a polynomial is given that can be used to calculate the heat capacity of the *P*2_1_/*c‐*polymorph at high temperatures. With the exception of the standard enthalpy of formation of K_4_CaSi_6_O_15_, the last thermodynamic key property that is still missing (it might be estimated or, more desirably, be determined experimentally in the future), a complete thermodynamic description of K_4_CaSi_6_O_15_ is now available allowing to incorporate this phase into thermodynamic modeling software used to compute and optimize existing phase diagrams for ceramic and slag systems. Apart from K_4_CaSi_6_O_15_, there are eight structurally characterized crystalline compounds in the K_2_O‐CaO‐SiO_2_ system at ambient conditions. It cannot be ruled out that they form polymorphs under nonambient conditions. In particular, K_9.6_CaSi_12_O_30_ could be an interesting candidate to consider, since it is also based on an interrupted silica framework and filled by channel‐shaped cavities containing partially occupied potassium sites. Due to the almost complete lack of data on the thermodynamics of the ternary compounds in the system K_2_O‐CaO‐SiO_2_, the synthesis of high‐purity compounds and using multiple thermal analysis techniques is an absolute necessity. The results of the thermal analyses can then serve as a starting point for investigating polymorphism. Finally, we would like to stress that the description of the diffraction features of the incommensurately modulated intermediate temperature phase is only the first step toward a complete structural characterization. The full structure solution and refinement using a superspace approach would require much better single‐crystal diffraction data sets. An in‐situ high‐temperature diffraction study using synchrotron radiation could provide sufficiently strong intensities for the weak satellite reflections, which carry the crucial information of the modulation.

## Supporting information

Supporting information

Supporting information

Supporting information

Supporting information
