# Supplementary material for: Investigations on the polymorphism of K4CaSi6O15 at elevated temperatures
Source: J Am Ceram Soc. 2023 Jul 13;106(11):7109–22. doi: 10.1111/jace.19310 (PMC10962641; doi:10.1111/jace.19310)
Supplement: Supplementary file 1 — Supporting information [file JACE-106-7109-s001.docx]

Figure S1. Temperature dependent evolution of the unit-cell parameters *a*, *b*, *c*, the monoclinic angle *β,* and the cell volume.
