# Supplementary material for: Investigations on the polymorphism of K4CaSi6O15 at elevated temperatures
Source: J Am Ceram Soc. 2023 Jul 13;106(11):7109–22. doi: 10.1111/jace.19310 (PMC10962641; doi:10.1111/jace.19310)
Supplement: Supplementary file 2 — Supporting information [file JACE-106-7109-s004.pdf]

Table S1. Heat capacity of $K_4CaSi_6O_{15}$ (M = 604.979 g/mol), synthesized in this study. Calorimetric data at various temperatures were obtained by relaxation calorimetry using a Physical Properties Measurement System (PPMS) and by Differential Scanning Calorimetry (DSC).

PPMS			DSC		
T (K)	Cp (J/mol.K)	sigma-Cp (J/mol.K)	T (K)	Cp (J/mol.K)	sigma-Cp (J/mol.K)
2.32859	0.0195718	0.000652955	284.234	443.719	2.49178
2.52118	0.0248495	0.000725339	288.705	447.04	2.01516
2.72972	0.0315781	0.000815098	293.187	450.502	1.91461
2.95308	0.0401401	0.000923261	297.674	453.98	1.90632
3.19298	0.0511527	0.00105369	302.168	457.311	1.75343
3.4572	0.0675262	0.00131244	306.668	460.535	1.47534
3.73801	0.0904317	0.00160852	311.176	463.635	1.2344
4.0445	0.123423	0.00178495	315.689	466.831	1.21627
4.37896	0.164767	0.00210061	320.963	470.477	1.10681
4.73997	0.218815	0.00247363	334.877	479.621	0.766666
5.13324	0.292085	0.00283706	339.411	482.436	0.81049
5.56491	0.391228	0.00333865	343.941	485.213	0.901655
6.05217	0.527007	0.00400632	348.473	487.841	1.03862
6.57364	0.7068	0.00483961	353.002	490.524	1.23071
7.13414	0.93254	0.00430752	357.531	493.18	1.41257
7.6703	1.18727	0.00655527	362.059	495.761	1.59021
8.32738	1.55168	0.00807986	366.588	498.52	1.6421
9.04218	2.01077	0.0100797	372.504	502.059	1.60995
9.82362	2.5915	0.0122217	385.034	509.354	2.25417
10.6798	3.3298	0.0148958	389.555	512.063	2.82241
11.6116	4.22907	0.017979	394.075	514.236	3.18446
12.6015	5.31571	0.0229978	398.593	517.046	2.84304
13.6912	6.65901	0.0270357	403.112	519.4	3.04214
14.8608	8.24051	0.0323275	407.628	521.783	3.24906
16.1431	10.2089	0.0397699	412.148	524.275	3.09983
17.5268	12.5143	0.0489449	416.663	526.814	2.93396
19.0581	15.4281	0.0637515	423.254	530.618	2.72487
20.7454	18.6377	0.0511775	435.03	537.619	2.58357
22.5421	22.7544	0.0842983	439.543	540.615	2.73545
24.4828	26.9737	0.0671633	444.053	545.01	2.94525
26.6144	32.3038	0.0824381	448.562	554.291	3.38173
28.9443	38.1789	0.0918268	453.066	566.929	3.65654
31.4636	44.8041	0.104027	457.576	582.864	4.60005
34.1959	52.3806	0.124396	462.084	607.756	3.85767
37.1656	60.8322	0.139083	466.594	596.679	5.45574
40.42	69.8239	0.158611	473.888	562.096	3.44077
43.9448	79.7195	0.182871	485.015	558.809	3.58564
47.7952	90.7568	0.207886	489.521	560.512	3.6347
51.9627	102.257	0.226796	494.026	562.235	3.54809
56.5048	114.414	0.259491	498.531	564.004	3.4881
61.4483	127.589	0.283005	503.036	565.851	3.45724
66.8199	141.299	0.301929	507.54	567.377	3.49139
72.6927	155.81	0.358047	512.042	568.761	3.33239
79.0418	171.78	0.395158	516.545	570.28	3.19667
85.9531	188.155	0.420008	524.556	572.401	2.99419
93.4893	204.295	0.461344	535.027	575.158	3.00589
101.665	221.323	0.506014	539.526	576.476	3.08633
110.591	238.227	0.546072	544.028	577.907	3.3367
120.203	255.376	0.562535	548.528	579.313	3.38776
130.742	273.494	0.564174	553.03	580.762	3.62076
142.227	291.407	0.606171	557.528	582.148	3.76119
154.711	308.7	0.639931	562.028	583.284	3.87609
168.286	327.765	0.66406	566.527	584.885	4.12734
183.026	347.574	0.700528	575.245	587.113	4.03715
199.062	366.664	0.752237	585.022	589.802	3.81042
216.528	385.677	0.774641	589.519	591.147	3.74248

235.495	404.706	0.80379	594.02	592.499	3.70852
256.154	423.496	0.828515	598.516	593.949	3.67573
278.619	443.662	0.944325	603.015	595.345	3.5877
303.067	461.261	0.925154	607.51	597.05	3.36497
			612.008	598.782	2.96085
			616.505	600.284	2.55703
			625.936	603.846	2.2046
			635.017	607.302	2.04559
			639.513	609.347	2.07836
			644.011	611.458	2.35494
			648.504	613.703	2.76356
			653.003	616.159	3.21117
			657.497	619.008	3.6943
			661.997	622.632	4.27078
			666.493	627.012	4.64744
			676.626	617.853	4.63734
			684.998	606.322	4.44078
			689.493	604.624	4.40573
			693.993	603.929	4.611
			698.489	603.444	4.60604
			702.991	603.117	4.52373
			707.492	603.488	4.64859
			711.992	603.396	4.64984
			716.492	603.407	4.88808
			727.316	603.571	4.89758
			734.98	603.881	4.49415
			739.479	604.148	4.42924
			743.98	604.533	4.14739
			748.476	604.865	3.61989
			752.972	604.851	3.09819
			757.468	605.191	3.39169
			761.964	605.118	3.19009
			766.466	605.263	3.36905
