# Supplementary material for: Investigations on the polymorphism of K4CaSi6O15 at elevated temperatures
Source: J Am Ceram Soc. 2023 Jul 13;106(11):7109–22. doi: 10.1111/jace.19310 (PMC10962641; doi:10.1111/jace.19310)
Supplement: Supplementary file 3 — Supporting information [file JACE-106-7109-s002.pdf]

Table S2. Differential scanning calorimetry data for the heating and cooling cycle in the temperature regions around two phase transitions.

1st transition			
Heating Temperature (°C)	Sample Heat Flow (mW)	Cooling Temperature (°C)	Sample Heat Flow (mW)
150.15	0.169	250.11	0.3997
150.15	0.168	250.11	0.3972
150.15	0.168	250.07	0.3932
150.15	0.1698	250	0.4193
150.15	0.1693	249.9	0.3972
150.16	0.1676	249.77	0.1954
150.16	0.168	249.63	-0.2221
150.15	0.1685	249.48	-0.7918
150.15	0.1684	249.33	-1.4105
150.16	0.1684	249.17	-2.0001
150.15	0.1682	249	-2.5158
150.15	0.1683	248.84	-2.9405
150.15	0.169	248.67	-3.2841
150.15	0.1705	248.51	-3.5531
150.15	0.1695	248.34	-3.7572
150.15	0.1696	248.18	-3.9134
150.15	0.1709	248.01	-4.034
150.15	0.1715	247.85	-4.1258
150.15	0.1722	247.68	-4.1949
150.15	0.1719	247.51	-4.2463
150.15	0.1725	247.35	-4.2838
150.15	0.1727	247.18	-4.3105
150.15	0.1727	247.01	-4.3316
150.15	0.1718	246.85	-4.3495
150.15	0.1718	246.68	-4.3619
150.15	0.1729	246.52	-4.3694
150.15	0.1739	246.35	-4.3764
150.15	0.1743	246.18	-4.3816
150.15	0.1745	246.02	-4.3851
150.15	0.1738	245.85	-4.3885
150.15	0.1724	245.69	-4.3912
150.15	0.1734	245.52	-4.3927
150.15	0.1746	245.35	-4.3952
150.15	0.1743	245.19	-4.3943
150.15	0.1728	245.02	-4.392
150.15	0.1721	244.85	-4.3922
150.15	0.1727	244.69	-4.3938
150.15	0.1752	244.52	-4.3954
150.15	0.177	244.35	-4.3957
150.15	0.1765	244.19	-4.3943
150.15	0.1771	244.02	-4.3954
150.15	0.177	243.85	-4.3971
150.15	0.1759	243.69	-4.3974
150.15	0.1758	243.52	-4.3956
150.15	0.1747	243.36	-4.3949
150.15	0.1748	243.19	-4.3946
150.15	0.1767	243.02	-4.3954

2nd transition			
Heating Temperature (°C)	Sample Heat Flow (mW)	Cooling Temperature (°C)	Sample Heat Flow (mW)
350.06	0.6949	450.01	1.1316
350.06	0.6953	450.01	1.1339
350.05	0.6945	449.97	1.1268
350.06	0.6948	449.9	1.152
350.06	0.696	449.78	1.1094
350.06	0.6982	449.64	0.8106
350.06	0.6978	449.5	0.2283
350.06	0.6962	449.34	-0.5179
350.06	0.6962	449.17	-1.268
350.06	0.6953	449.01	-1.9241
350.06	0.6949	448.85	-2.4532
350.06	0.6938	448.68	-2.8601
350.06	0.6941	448.51	-3.1634
350.06	0.6943	448.34	-3.3885
350.05	0.6964	448.18	-3.5519
350.06	0.6987	448.01	-3.6721
350.06	0.6999	447.85	-3.7574
350.06	0.6975	447.68	-3.8157
350.06	0.6956	447.52	-3.8601
350.06	0.6936	447.35	-3.8996
350.06	0.6918	447.18	-3.9277
350.06	0.692	447.02	-3.9414
350.06	0.6945	446.85	-3.9487
350.06	0.6957	446.68	-3.9545
350.06	0.6963	446.52	-3.9633
350.05	0.6962	446.35	-3.9686
350.06	0.6962	446.19	-3.9708
350.06	0.6964	446.02	-3.9763
350.06	0.6982	445.85	-3.9799
350.06	0.6997	445.69	-3.9819
350.06	0.6994	445.52	-3.9814
350.06	0.6948	445.35	-3.9833
350.06	0.6934	445.19	-3.9833
350.06	0.6937	445.02	-3.9838
350.06	0.6943	444.85	-3.9867
350.06	0.695	444.69	-3.9865
350.06	0.6959	444.52	-3.9862
350.06	0.6984	444.35	-3.9897
350.06	0.7012	444.19	-3.9922
350.06	0.7013	444.02	-3.9924
350.06	0.6972	443.85	-3.9938
350.06	0.6956	443.69	-3.9974
350.06	0.6967	443.52	-3.999
350.06	0.6955	443.36	-4.0009
350.06	0.6927	443.19	-4.001
350.06	0.6924	443.02	-3.9995
350.06	0.6938	442.86	-3.9983

150.15	0.1775	242.85	-4.3983
150.15	0.1775	242.68	-4.3975
150.15	0.1775	242.52	-4.3969
150.15	0.1765	242.35	-4.3971
150.15	0.1762	242.19	-4.3962
150.15	0.1773	242.02	-4.3977
150.15	0.1775	241.85	-4.3996
150.15	0.1784	241.69	-4.3991
150.15	0.1789	241.52	-4.398
150.15	0.1792	241.35	-4.3975
150.15	0.1799	241.19	-4.3967
150.15	0.1794	241.02	-4.3965
150.15	0.1786	240.86	-4.3973
150.15	0.1788	240.69	-4.3979
150.15	0.1794	240.52	-4.3975
150.15	0.1799	240.36	-4.3953
150.15	0.1809	240.19	-4.3932
150.15	0.1805	240.02	-4.3943
150.15	0.1807	239.86	-4.396
150.15	0.1811	239.69	-4.3958
150.15	0.1793	239.52	-4.3962
150.15	0.1795	239.36	-4.3954
150.15	0.1799	239.19	-4.3951
150.15	0.1796	239.02	-4.3953
150.15	0.1799	238.85	-4.396
150.15	0.1802	238.69	-4.396
150.15	0.1792	238.52	-4.3958
150.15	0.18	238.35	-4.3961
150.15	0.1799	238.19	-4.3944
150.15	0.1791	238.02	-4.3963
150.15	0.179	237.86	-4.4005
150.15	0.179	237.69	-4.4015
150.15	0.1795	237.52	-4.4004
150.15	0.1798	237.36	-4.3992
150.15	0.1793	237.19	-4.3984
150.15	0.1803	237.02	-4.3978
150.15	0.1811	236.85	-4.3985
150.15	0.1808	236.69	-4.3997
150.15	0.1797	236.52	-4.3991
150.15	0.1802	236.35	-4.3992
150.15	0.1804	236.19	-4.3988
150.15	0.1803	236.02	-4.3973
150.15	0.1798	235.86	-4.3948
150.15	0.1798	235.69	-4.3936
150.15	0.1792	235.52	-4.3935
150.15	0.1785	235.36	-4.3947
150.15	0.1788	235.19	-4.3941
150.15	0.1805	235.02	-4.3916
150.15	0.1817	234.86	-4.3916
150.15	0.1817	234.69	-4.391
150.15	0.181	234.53	-4.3909

350.06	0.6968	442.69	-3.9979
350.06	0.699	442.52	-3.9974
350.06	0.7025	442.36	-3.9931
350.06	0.7042	442.19	-3.9916
350.06	0.7033	442.02	-3.9945
350.06	0.7029	441.86	-4.0011
350.06	0.6996	441.69	-4.0025
350.06	0.6991	441.52	-4.0048
350.06	0.6993	441.36	-4.0061
350.06	0.6994	441.19	-4.0081
350.06	0.6986	441.03	-4.0086
350.06	0.6985	440.86	-4.003
350.06	0.6956	440.69	-4.0009
350.06	0.6958	440.53	-4.0072
350.06	0.6988	440.37	-4.0084
350.06	0.699	440.19	-4.0055
350.06	0.6971	440.03	-4.0012
350.06	0.6975	439.86	-3.9999
350.06	0.6981	439.69	-4.0017
350.06	0.6981	439.53	-4.0057
350.06	0.6937	439.36	-4.0067
350.06	0.6903	439.19	-4.002
350.06	0.6911	439.03	-4.0032
350.06	0.695	438.86	-4.0042
350.06	0.6995	438.69	-4.0073
350.06	0.7015	438.52	-4.0095
350.06	0.7015	438.36	-4.0131
350.06	0.6998	438.19	-4.0187
350.06	0.7002	438.03	-4.0211
350.06	0.7015	437.86	-4.0192
350.06	0.7027	437.69	-4.0154
350.06	0.7027	437.52	-4.013
350.06	0.7028	437.36	-4.0139
350.06	0.7017	437.19	-4.0178
350.06	0.7044	437.03	-4.0186
350.06	0.7028	436.86	-4.0187
350.06	0.6976	436.69	-4.0182
350.06	0.6962	436.53	-4.0198
350.06	0.6974	436.36	-4.0202
350.06	0.6942	436.19	-4.0213
350.06	0.6938	436.02	-4.0196
350.06	0.6967	435.86	-4.0177
350.06	0.6982	435.69	-4.0191
350.06	0.6998	435.53	-4.0202
350.06	0.6996	435.37	-4.0226
350.06	0.6968	435.2	-4.0284
350.06	0.6962	435.03	-4.0294
350.06	0.6962	434.86	-4.0287
350.06	0.6981	434.7	-4.0293
350.06	0.6989	434.53	-4.029
350.06	0.6974	434.37	-4.0318

150.15	0.1804	234.36	-4.3939
150.15	0.181	234.19	-4.398
150.14	0.1804	234.02	-4.3982
150.15	0.1786	233.86	-4.3977
150.15	0.1781	233.69	-4.3987
150.14	0.1806	233.53	-4.3987
150.15	0.1819	233.36	-4.3999
150.15	0.1817	233.19	-4.3999
150.15	0.1821	233.03	-4.4
150.14	0.1823	232.86	-4.4012
150.15	0.1816	232.7	-4.4024
150.15	0.1807	232.53	-4.4018
150.14	0.18	232.36	-4.4007
150.14	0.1799	232.19	-4.3995
150.14	0.1792	232.03	-4.3986
150.15	0.1799	231.86	-4.4008
150.15	0.1801	231.69	-4.4012
150.14	0.1798	231.53	-4.4004
150.15	0.1793	231.36	-4.3997
150.14	0.1801	231.19	-4.3991
150.14	0.1797	231.03	-4.3978
150.14	0.1801	230.86	-4.3975
150.15	0.1809	230.69	-4.3974
150.14	0.1816	230.53	-4.3963
150.14	0.1818	230.36	-4.3968
150.14	0.1809	230.19	-4.3982
150.15	0.1804	230.03	-4.3981
150.14	0.1805	229.86	-4.3996
150.15	0.1803	229.69	-4.4014
150.15	0.1803	229.53	-4.4007
150.15	0.18	229.36	-4.3976
150.15	0.1798	229.19	-4.3979
150.15	0.1796	229.03	-4.3998
150.14	0.1798	228.86	-4.4015
150.14	0.18	228.69	-4.4017
150.14	0.1798	228.53	-4.4017
150.14	0.1795	228.36	-4.4016
150.14	0.1802	228.19	-4.402
150.14	0.1802	228.02	-4.4022
150.14	0.1806	227.86	-4.4024
150.14	0.1799	227.69	-4.4024
150.15	0.179	227.53	-4.4027
150.15	0.1791	227.36	-4.402
150.15	0.1794	227.19	-4.4027
150.15	0.1797	227.03	-4.4025
150.15	0.1812	226.86	-4.4039
150.15	0.1827	226.69	-4.4056
150.15	0.1816	226.53	-4.4057
150.15	0.1815	226.36	-4.4037
150.15	0.1836	226.19	-4.4035
150.14	0.1833	226.03	-4.4032

350.06	0.6965	434.2	-4.0385
350.06	0.6949	434.03	-4.0386
350.06	0.6943	433.86	-4.0352
350.06	0.6945	433.69	-4.0358
350.06	0.6945	433.53	-4.0382
350.06	0.6934	433.36	-4.0407
350.06	0.6961	433.2	-4.0404
350.06	0.6974	433.03	-4.0411
350.06	0.6963	432.86	-4.0435
350.06	0.6932	432.69	-4.0438
350.06	0.6936	432.53	-4.0444
350.06	0.6945	432.36	-4.0448
350.06	0.694	432.2	-4.0448
350.06	0.6955	432.03	-4.0487
350.06	0.6972	431.87	-4.0505
350.06	0.6975	431.7	-4.0509
350.06	0.6985	431.53	-4.0521
350.06	0.6973	431.36	-4.0564
350.06	0.6966	431.2	-4.0586
350.06	0.6967	431.03	-4.062
350.06	0.6953	430.87	-4.0602
350.06	0.6949	430.7	-4.0609
350.06	0.6936	430.53	-4.0614
350.06	0.6939	430.37	-4.0632
350.06	0.6967	430.2	-4.0662
350.06	0.6966	430.03	-4.0677
350.06	0.6965	429.86	-4.0734
350.06	0.6987	429.7	-4.074
350.06	0.6989	429.53	-4.0735
350.06	0.6975	429.36	-4.0702
350.06	0.6964	429.2	-4.069
350.06	0.696	429.03	-4.0735
350.06	0.6947	428.87	-4.0751
350.06	0.6975	428.7	-4.0733
350.06	0.6981	428.53	-4.0744
350.06	0.6966	428.37	-4.0716
350.06	0.6975	428.2	-4.0696
350.06	0.6968	428.03	-4.0748
350.06	0.6945	427.86	-4.0784
350.06	0.6918	427.69	-4.0798
350.06	0.6922	427.53	-4.0807
350.06	0.6949	427.36	-4.0785
350.06	0.6929	427.2	-4.0719
350.06	0.687	427.03	-4.0719
350.06	0.6846	426.86	-4.076
350.06	0.6907	426.69	-4.0778
350.06	0.6942	426.53	-4.0808
350.06	0.6969	426.36	-4.0803
350.06	0.6969	426.2	-4.08
350.06	0.6956	426.03	-4.0799
350.06	0.6983	425.86	-4.0795

150.14	0.1823	225.86	-4.4008
150.14	0.1809	225.69	-4.4011
150.15	0.1791	225.53	-4.4013
150.15	0.1802	225.36	-4.4024
150.14	0.1824	225.19	-4.4055
150.15	0.1829	225.03	-4.4068
150.14	0.1829	224.86	-4.4078
150.14	0.1835	224.69	-4.4096
150.14	0.1838	224.53	-4.4102
150.14	0.1835	224.36	-4.4089
150.14	0.1835	224.2	-4.4084
150.14	0.1834	224.03	-4.4086
150.14	0.1835	223.86	-4.4082
150.14	0.1832	223.7	-4.4087
150.14	0.1836	223.53	-4.4095
150.15	0.1837	223.36	-4.4077
150.14	0.1831	223.2	-4.4072
150.14	0.1827	223.03	-4.4073
150.14	0.1823	222.86	-4.4063
150.14	0.1829	222.7	-4.4066
150.14	0.1841	222.53	-4.4058
150.14	0.1844	222.36	-4.4037
150.14	0.1839	222.19	-4.4035
150.14	0.1828	222.03	-4.4041
150.14	0.1836	221.86	-4.404
150.14	0.1834	221.69	-4.4028
150.14	0.1824	221.53	-4.4022
150.14	0.1834	221.36	-4.4026
150.14	0.1849	221.19	-4.4032
150.14	0.1842	221.02	-4.4037
150.14	0.1837	220.86	-4.4038
150.14	0.1839	220.69	-4.4046
150.15	0.1861	220.53	-4.406
150.18	0.1905	220.36	-4.4052
150.24	0.1669	220.19	-4.4057
150.34	0.172	220.02	-4.4067
150.46	0.3189	219.86	-4.4051
150.6	0.6495	219.69	-4.4046
150.74	1.1218	219.53	-4.4047
150.9	1.6642	219.36	-4.4057
151.06	2.1997	219.19	-4.4063
151.22	2.6784	219.02	-4.407
151.38	3.088	218.86	-4.4062
151.54	3.4277	218.69	-4.4051
151.71	3.7043	218.52	-4.4036
151.87	3.9266	218.36	-4.4035
152.04	4.1032	218.19	-4.4041
152.21	4.242	218.03	-4.4045
152.37	4.3495	217.86	-4.4042
152.54	4.4318	217.69	-4.405
152.71	4.4969	217.53	-4.4059

350.06	0.6998	425.7	-4.0856
350.06	0.6989	425.53	-4.0917
350.06	0.6986	425.36	-4.0896
350.06	0.6973	425.19	-4.0903
350.06	0.6955	425.03	-4.0914
350.06	0.6949	424.87	-4.0917
350.06	0.6927	424.7	-4.0959
350.06	0.692	424.54	-4.0974
350.06	0.6942	424.37	-4.0949
350.06	0.6954	424.2	-4.0906
350.06	0.6954	424.03	-4.0907
350.06	0.6944	423.87	-4.0939
350.06	0.6941	423.7	-4.0935
350.06	0.6934	423.54	-4.0934
350.06	0.6935	423.37	-4.0969
350.06	0.6933	423.2	-4.0968
350.06	0.6941	423.03	-4.0973
350.06	0.6949	422.87	-4.1003
350.06	0.6954	422.7	-4.1019
350.06	0.6937	422.54	-4.107
350.06	0.6949	422.37	-4.1068
350.06	0.6942	422.2	-4.1053
350.06	0.6936	422.04	-4.1068
350.06	0.6951	421.87	-4.1057
350.06	0.6951	421.7	-4.1046
350.06	0.695	421.53	-4.1066
350.06	0.6953	421.37	-4.1081
350.06	0.6948	421.2	-4.1102
350.06	0.6978	421.04	-4.1136
350.06	0.6994	420.87	-4.1136
350.06	0.6976	420.7	-4.1138
350.06	0.6941	420.54	-4.1168
350.07	0.6956	420.37	-4.1164
350.1	0.6997	420.21	-4.1171
350.17	0.6724	420.04	-4.1148
350.28	0.725	419.87	-4.1143
350.42	1.0056	419.7	-4.1151
350.57	1.537	419.54	-4.1148
350.72	2.2213	419.37	-4.1197
350.88	2.9304	419.2	-4.1215
351.05	3.5773	419.04	-4.122
351.21	4.1154	418.88	-4.1239
351.38	4.5391	418.71	-4.1238
351.54	4.8613	418.54	-4.125
351.71	5.1044	418.38	-4.1288
351.87	5.294	418.21	-4.129
352.04	5.4372	418.04	-4.13
352.2	5.5411	417.87	-4.1314
352.37	5.6135	417.71	-4.1317
352.54	5.6711	417.54	-4.1339
352.71	5.7147	417.37	-4.1362

152.87	4.5485	217.36	-4.4045
153.04	4.5879	217.19	-4.4045
153.21	4.6167	217.03	-4.4039
153.37	4.6385	216.86	-4.4029
153.54	4.6573	216.7	-4.4004
153.71	4.6725	216.53	-4.3999
153.88	4.6804	216.36	-4.4004
154.04	4.6861	216.19	-4.4018
154.21	4.6938	216.03	-4.403
154.38	4.7009	215.86	-4.4031
154.55	4.7069	215.69	-4.4039
154.72	4.711	215.53	-4.4058
154.88	4.7143	215.36	-4.4055
155.05	4.7162	215.19	-4.4026
155.21	4.7167	215.02	-4.4005
155.38	4.7195	214.86	-4.3998
155.55	4.7242	214.69	-4.3989
155.72	4.7271	214.53	-4.3999
155.88	4.7273	214.36	-4.4016
156.06	4.7278	214.19	-4.401
156.22	4.7305	214.03	-4.3995
156.39	4.7314	213.86	-4.3987
156.55	4.7327	213.69	-4.3989
156.72	4.7335	213.53	-4.3998
156.89	4.7345	213.36	-4.4004
157.06	4.7356	213.19	-4.3985
157.23	4.7371	213.03	-4.3963
157.39	4.74	212.86	-4.3968
157.56	4.7425	212.69	-4.3988
157.73	4.7459	212.52	-4.401
157.89	4.7471	212.36	-4.4002
158.06	4.7472	212.19	-4.3997
158.23	4.7474	212.03	-4.4007
158.39	4.7481	211.86	-4.4008
158.56	4.7484	211.69	-4.4017
158.73	4.7479	211.52	-4.4002
158.9	4.7482	211.36	-4.399
159.06	4.7492	211.19	-4.3969
159.23	4.7491	211.03	-4.3957
159.4	4.7488	210.86	-4.3962
159.56	4.7495	210.69	-4.3973
159.73	4.7513	210.53	-4.3975
159.9	4.7526	210.36	-4.3973
160.07	4.7532	210.19	-4.3954
160.24	4.7538	210.02	-4.3944
160.4	4.7541	209.86	-4.3943
160.57	4.7563	209.69	-4.3941
160.74	4.7591	209.52	-4.3928
160.91	4.7598	209.36	-4.392
161.07	4.7611	209.19	-4.3919
161.24	4.7627	209.02	-4.3926

352.87	5.7431	417.21	-4.1354
353.04	5.7604	417.04	-4.1366
353.21	5.7741	416.87	-4.1373
353.37	5.7859	416.7	-4.1393
353.54	5.7945	416.54	-4.1414
353.7	5.7999	416.37	-4.1429
353.87	5.807	416.21	-4.1454
354.04	5.8123	416.04	-4.1429
354.2	5.8158	415.87	-4.1401
354.37	5.8182	415.71	-4.1429
354.54	5.8236	415.54	-4.1428
354.7	5.8286	415.38	-4.1421
354.87	5.8293	415.21	-4.1446
355.04	5.8298	415.05	-4.1477
355.2	5.8322	414.88	-4.1471
355.37	5.8332	414.71	-4.1475
355.54	5.8349	414.54	-4.1475
355.7	5.8366	414.38	-4.146
355.87	5.837	414.22	-4.1469
356.03	5.8354	414.05	-4.1504
356.2	5.8373	413.88	-4.1518
356.37	5.8381	413.72	-4.1526
356.54	5.8388	413.55	-4.1552
356.7	5.8404	413.38	-4.1582
356.87	5.844	413.21	-4.1589
357.04	5.8439	413.05	-4.1598
357.2	5.8432	412.88	-4.1608
357.37	5.8434	412.72	-4.16
357.54	5.8479	412.55	-4.1618
357.7	5.853	412.38	-4.1685
357.87	5.8535	412.22	-4.1735
358.04	5.856	412.05	-4.1741
358.2	5.8545	411.88	-4.1732
358.37	5.8588	411.72	-4.1709
358.54	5.8591	411.55	-4.1699
358.7	5.8598	411.38	-4.1722
358.87	5.8608	411.22	-4.1721
359.04	5.8618	411.05	-4.1704
359.2	5.8648	410.88	-4.1709
359.37	5.8682	410.72	-4.1741
359.54	5.8666	410.55	-4.1768
359.7	5.865	410.38	-4.1789
359.87	5.8646	410.22	-4.1829
360.04	5.8635	410.05	-4.1827
360.21	5.8646	409.88	-4.1866
360.38	5.8656	409.72	-4.1865
360.54	5.8618	409.55	-4.1886
360.7	5.8622	409.38	-4.1896
360.87	5.8671	409.22	-4.1917
361.04	5.8748	409.05	-4.1923
361.2	5.8798	408.88	-4.194

161.4	4.7629	208.85	-4.3944
161.57	4.763	208.69	-4.3947
161.74	4.765	208.52	-4.3938
161.91	4.7659	208.36	-4.3933
162.07	4.7669	208.19	-4.3935
162.24	4.7677	208.02	-4.3945
162.4	4.7663	207.86	-4.3964
162.57	4.7675	207.69	-4.3974
162.74	4.7706	207.53	-4.3983
162.91	4.7717	207.36	-4.3972
163.07	4.7704	207.19	-4.3948
163.24	4.7699	207.03	-4.3928
163.41	4.771	206.86	-4.3931
163.57	4.7737	206.69	-4.3931
163.74	4.7755	206.53	-4.3925
163.91	4.7772	206.36	-4.392
164.08	4.7776	206.19	-4.3918
164.24	4.7779	206.03	-4.3909
164.41	4.78	205.86	-4.3898
164.58	4.782	205.69	-4.3887
164.75	4.7834	205.53	-4.3878
164.92	4.7833	205.36	-4.3884
165.08	4.7857	205.2	-4.3878
165.25	4.788	205.03	-4.3876
165.41	4.789	204.86	-4.3877
165.59	4.7896	204.69	-4.3891
165.75	4.7901	204.52	-4.3883
165.92	4.7913	204.36	-4.387
166.08	4.7941	204.19	-4.3857
166.25	4.7949	204.03	-4.386
166.42	4.7957	203.86	-4.3882
166.59	4.7974	203.69	-4.3881
166.75	4.7991	203.53	-4.389
166.92	4.8001	203.36	-4.3894
167.08	4.8004	203.19	-4.3905
167.25	4.802	203.02	-4.3901
167.42	4.8019	202.86	-4.3902
167.59	4.801	202.69	-4.3907
167.75	4.8026	202.53	-4.3904
167.92	4.8047	202.36	-4.39
168.09	4.8071	202.19	-4.3904
168.25	4.8084	202.03	-4.3901
168.42	4.8089	201.86	-4.3896
168.59	4.8118	201.7	-4.3874
168.75	4.8135	201.53	-4.3853
168.92	4.8158	201.36	-4.3851
169.09	4.8177	201.19	-4.3855
169.26	4.8198	201.03	-4.3864
169.42	4.8227	200.86	-4.3859
169.59	4.8246	200.69	-4.3872
169.76	4.8271	200.52	-4.3883

361.37	5.8846	408.71	-4.1972
361.54	5.887	408.55	-4.1982
361.7	5.8859	408.38	-4.1963
361.87	5.8863	408.22	-4.1998
362.04	5.8875	408.05	-4.2048
362.2	5.891	407.89	-4.2075
362.37	5.8938	407.72	-4.2099
362.54	5.8918	407.55	-4.2125
362.7	5.8886	407.38	-4.2139
362.87	5.8887	407.22	-4.2159
363.03	5.8908	407.05	-4.2157
363.2	5.893	406.89	-4.2171
363.37	5.8932	406.72	-4.2207
363.54	5.8932	406.55	-4.2198
363.7	5.8913	406.38	-4.2209
363.87	5.8922	406.21	-4.2249
364.04	5.8952	406.05	-4.2294
364.2	5.8971	405.88	-4.2299
364.37	5.8998	405.72	-4.2306
364.53	5.9036	405.55	-4.2324
364.7	5.9062	405.39	-4.2357
364.87	5.9062	405.22	-4.241
365.04	5.9047	405.05	-4.2422
365.2	5.9049	404.88	-4.2411
365.37	5.9095	404.72	-4.2439
365.53	5.913	404.55	-4.2484
365.7	5.9116	404.39	-4.2508
365.87	5.9117	404.22	-4.2537
366.04	5.9135	404.06	-4.2556
366.2	5.9146	403.89	-4.2586
366.37	5.9115	403.72	-4.262
366.54	5.9093	403.55	-4.2612
366.7	5.9145	403.39	-4.2633
366.87	5.9277	403.22	-4.2693
367.04	5.9331	403.06	-4.271
367.2	5.9328	402.89	-4.2754
367.37	5.9302	402.72	-4.28
367.54	5.9262	402.56	-4.2815
367.7	5.9242	402.39	-4.2842
367.87	5.9244	402.23	-4.2864
368.03	5.9254	402.06	-4.2887
368.2	5.9304	401.89	-4.2908
368.37	5.9338	401.73	-4.2916
368.53	5.9329	401.56	-4.2964
368.7	5.932	401.39	-4.3042
368.87	5.9311	401.23	-4.3115
369.03	5.9299	401.06	-4.3146
369.2	5.9293	400.89	-4.3159
369.37	5.9291	400.73	-4.3188
369.53	5.933	400.56	-4.3237
369.7	5.9335	400.39	-4.3314

169.92	4.8289	200.36	-4.3881
170.09	4.8315	200.19	-4.3871
170.26	4.8326	200.02	-4.3844
170.42	4.8349	199.86	-4.3849
170.59	4.8359	199.69	-4.3848
170.76	4.8373	199.52	-4.385
170.92	4.8401	199.36	-4.3853
171.09	4.8413	199.19	-4.3848
171.26	4.8416	199.02	-4.3837
171.43	4.8446	198.86	-4.3835
171.59	4.8473	198.69	-4.3851
171.76	4.8491	198.52	-4.3863
171.93	4.8494	198.35	-4.3868
172.09	4.8492	198.19	-4.3867
172.26	4.849	198.02	-4.386
172.43	4.8532	197.86	-4.3857
172.6	4.8575	197.69	-4.3857
172.77	4.861	197.52	-4.3847
172.93	4.8634	197.36	-4.3845
173.1	4.8677	197.19	-4.3834
173.26	4.8718	197.02	-4.3822
173.43	4.8744	196.86	-4.3821
173.6	4.8767	196.69	-4.3828
173.77	4.8798	196.53	-4.3813
173.93	4.8814	196.36	-4.3803
174.1	4.8831	196.19	-4.3808
174.26	4.8847	196.03	-4.3801
174.43	4.8876	195.86	-4.3804
174.6	4.8909	195.69	-4.3813
174.77	4.8941	195.53	-4.3812
174.93	4.899	195.36	-4.3812
175.1	4.9041	195.19	-4.3817
175.27	4.9081	195.02	-4.3813
175.43	4.9125	194.86	-4.3818
175.6	4.9178	194.69	-4.3823
175.77	4.9216	194.52	-4.382
175.93	4.9243	194.36	-4.3814
176.1	4.9244	194.19	-4.3813
176.27	4.9261	194.03	-4.3795
176.44	4.9314	193.86	-4.3796
176.6	4.9377	193.69	-4.3818
176.77	4.9428	193.52	-4.3804
176.94	4.9449	193.35	-4.3778
177.1	4.9495	193.19	-4.3773
177.27	4.9532	193.02	-4.377
177.44	4.9587	192.86	-4.3762
177.61	4.963	192.69	-4.3754
177.77	4.9668	192.52	-4.3751
177.94	4.9718	192.36	-4.3742
178.1	4.9744	192.19	-4.3761
178.27	4.9779	192.03	-4.3761

369.87	5.9367	400.23	-4.3375
370.03	5.9404	400.06	-4.3429
370.2	5.944	399.89	-4.3481
370.36	5.9455	399.73	-4.3521
370.53	5.9492	399.56	-4.3597
370.7	5.9479	399.39	-4.3691
370.87	5.9483	399.23	-4.3727
371.04	5.9483	399.06	-4.3794
371.2	5.947	398.89	-4.3837
371.37	5.9436	398.73	-4.3866
371.53	5.9439	398.56	-4.3917
371.7	5.9531	398.4	-4.3968
371.86	5.9623	398.23	-4.4007
372.03	5.9689	398.06	-4.4067
372.2	5.9709	397.9	-4.4119
372.37	5.9691	397.73	-4.4223
372.53	5.9693	397.56	-4.4316
372.69	5.969	397.4	-4.4393
372.86	5.967	397.23	-4.4452
373.03	5.9657	397.06	-4.4502
373.2	5.9634	396.9	-4.4527
373.36	5.9632	396.73	-4.4558
373.53	5.9671	396.56	-4.4571
373.69	5.9735	396.39	-4.4628
373.86	5.9803	396.23	-4.4683
374.03	5.9792	396.06	-4.4765
374.19	5.9744	395.89	-4.4858
374.36	5.9741	395.73	-4.4923
374.53	5.9776	395.56	-4.4936
374.69	5.981	395.39	-4.4945
374.86	5.9838	395.23	-4.4969
375.02	5.9849	395.06	-4.4991
375.19	5.9866	394.9	-4.5024
375.35	5.9934	394.73	-4.5014
375.52	5.9948	394.56	-4.4986
375.69	5.9937	394.39	-4.496
375.86	5.996	394.23	-4.4981
376.02	5.9969	394.06	-4.5011
376.19	5.997	393.9	-4.4976
376.36	5.9972	393.73	-4.4954
376.53	5.9965	393.57	-4.4947
376.69	5.9973	393.4	-4.4949
376.86	6.0008	393.24	-4.4923
377.02	6.0007	393.07	-4.4902
377.19	6.0007	392.9	-4.4943
377.36	6.0036	392.74	-4.4975
377.52	6.0062	392.57	-4.4966
377.69	6.0094	392.4	-4.4956
377.86	6.014	392.24	-4.4943
378.02	6.0152	392.07	-4.492
378.19	6.0112	391.9	-4.4891

178.44	4.9829	191.86	-4.376
178.61	4.9877	191.69	-4.3751
178.77	4.9917	191.52	-4.3737
178.94	4.9947	191.35	-4.374
179.1	4.9974	191.19	-4.376
179.27	5.0007	191.02	-4.3772
179.43	5.0044	190.85	-4.3778
179.61	5.0083	190.69	-4.3771
179.77	5.0125	190.52	-4.3779
179.94	5.0183	190.35	-4.378
180.1	5.0234	190.19	-4.3792
180.27	5.027	190.02	-4.3797
180.44	5.0307	189.85	-4.379
180.6	5.0349	189.69	-4.3782
180.77	5.0386	189.52	-4.3776
180.94	5.0424	189.36	-4.376
181.1	5.046	189.19	-4.3739
181.27	5.0508	189.02	-4.3732
181.43	5.0547	188.86	-4.3732
181.6	5.0593	188.69	-4.3718
181.77	5.0654	188.52	-4.372
181.94	5.0709	188.35	-4.3726
182.11	5.0757	188.19	-4.3714
182.28	5.0791	188.02	-4.3707
182.44	5.0825	187.85	-4.3711
182.61	5.0874	187.69	-4.3723
182.78	5.0935	187.52	-4.372
182.95	5.0997	187.35	-4.3712
183.12	5.1055	187.19	-4.3714
183.28	5.1109	187.02	-4.372
183.45	5.1156	186.85	-4.37
183.61	5.1195	186.68	-4.3703
183.78	5.1243	186.52	-4.3719
183.94	5.1273	186.35	-4.3714
184.11	5.1317	186.19	-4.3714
184.28	5.1384	186.02	-4.3731
184.45	5.1444	185.85	-4.3725
184.62	5.1503	185.69	-4.3713
184.78	5.1562	185.52	-4.3703
184.95	5.1628	185.35	-4.368
185.11	5.169	185.19	-4.3661
185.28	5.1766	185.02	-4.3671
185.45	5.1833	184.85	-4.3666
185.62	5.1888	184.69	-4.3667
185.78	5.1935	184.52	-4.3679
185.95	5.1999	184.35	-4.3674
186.12	5.208	184.19	-4.3667
186.28	5.2195	184.02	-4.3643
186.45	5.2287	183.85	-4.3624
186.62	5.2352	183.69	-4.3615
186.78	5.2433	183.52	-4.3614

378.36	6.0082	391.74	-4.4846
378.52	6.0047	391.57	-4.4822
378.69	6.0048	391.41	-4.4815
378.86	6.0096	391.24	-4.4836
379.03	6.0148	391.07	-4.4847
379.19	6.0205	390.91	-4.4832
379.36	6.0229	390.74	-4.4815
379.53	6.0268	390.57	-4.4781
379.69	6.0278	390.41	-4.4767
379.86	6.0259	390.24	-4.478
380.02	6.0246	390.07	-4.4761
380.19	6.0287	389.9	-4.4695
380.36	6.034	389.74	-4.4632
380.52	6.0372	389.57	-4.451
380.69	6.0363	389.4	-4.4454
380.85	6.0362	389.24	-4.4487
381.02	6.0382	389.07	-4.452
381.19	6.0406	388.91	-4.4529
381.36	6.0436	388.74	-4.456
381.52	6.0445	388.57	-4.4576
381.69	6.0422	388.41	-4.4586
381.86	6.0325	388.24	-4.4584
382.02	6.0312	388.07	-4.4573
382.19	6.0371	387.91	-4.4587
382.35	6.0451	387.74	-4.4621
382.52	6.0518	387.57	-4.4635
382.69	6.0572	387.41	-4.4595
382.86	6.06	387.24	-4.456
383.02	6.0624	387.07	-4.453
383.19	6.0637	386.91	-4.4526
383.35	6.0655	386.74	-4.4491
383.52	6.0666	386.57	-4.4488
383.69	6.067	386.41	-4.4469
383.86	6.0687	386.24	-4.436
384.02	6.0682	386.07	-4.4326
384.19	6.066	385.91	-4.4389
384.35	6.0696	385.74	-4.4431
384.52	6.074	385.58	-4.4458
384.68	6.0768	385.41	-4.4483
384.85	6.0793	385.24	-4.4492
385.02	6.0784	385.08	-4.4473
385.18	6.0769	384.91	-4.4467
385.35	6.0795	384.74	-4.4462
385.52	6.0829	384.57	-4.4441
385.68	6.0867	384.41	-4.4388
385.85	6.0877	384.24	-4.4373
386.01	6.0887	384.08	-4.4404
386.19	6.0915	383.91	-4.4374
386.35	6.0932	383.74	-4.4328
386.52	6.0946	383.58	-4.4345
386.68	6.0979	383.41	-4.4407

186.95	5.2523	183.35	-4.3618
187.12	5.2621	183.19	-4.3617
187.28	5.27	183.02	-4.3616
187.45	5.2786	182.85	-4.3634
187.62	5.2877	182.68	-4.3643
187.79	5.2984	182.52	-4.3636
187.96	5.3091	182.35	-4.3626
188.12	5.319	182.18	-4.3625
188.29	5.329	182.01	-4.3622
188.45	5.3404	181.85	-4.363
188.62	5.3507	181.68	-4.3635
188.79	5.3603	181.51	-4.3636
188.95	5.3727	181.34	-4.3638
189.12	5.3842	181.18	-4.3608
189.29	5.395	181.01	-4.3594
189.46	5.4075	180.85	-4.3588
189.62	5.4194	180.68	-4.3614
189.79	5.43	180.51	-4.3619
189.96	5.4391	180.35	-4.3614
190.12	5.4484	180.18	-4.3601
190.29	5.4591	180.01	-4.3594
190.45	5.4697	179.85	-4.3595
190.62	5.4783	179.68	-4.3605
190.79	5.4861	179.51	-4.359
190.95	5.4933	179.35	-4.3583
191.12	5.4987	179.18	-4.3574
191.29	5.5029	179.01	-4.3569
191.46	5.5045	178.85	-4.3577
191.62	5.5052	178.68	-4.358
191.79	5.5057	178.52	-4.3579
191.96	5.5026	178.35	-4.3571
192.12	5.4981	178.18	-4.3559
192.29	5.4911	178.02	-4.3547
192.46	5.4825	177.85	-4.3551
192.63	5.4706	177.69	-4.353
192.79	5.4567	177.52	-4.3531
192.96	5.4426	177.35	-4.3546
193.13	5.4294	177.18	-4.355
193.29	5.415	177.01	-4.3539
193.46	5.3985	176.85	-4.3535
193.63	5.3808	176.68	-4.3538
193.8	5.3604	176.51	-4.3532
193.97	5.3409	176.35	-4.3543
194.13	5.3216	176.18	-4.3538
194.3	5.3041	176.01	-4.3541
194.47	5.2887	175.85	-4.3542
194.63	5.2721	175.68	-4.3544
194.8	5.2524	175.51	-4.3559
194.97	5.2341	175.35	-4.3555
195.13	5.2161	175.19	-4.3533
195.3	5.1979	175.02	-4.3527

386.85	6.0979	383.24	-4.4516
387.01	6.0969	383.08	-4.4635
387.18	6.0987	382.92	-4.4603
387.35	6.1014	382.75	-4.4505
387.52	6.1046	382.58	-4.441
387.69	6.1117	382.42	-4.4358
387.86	6.1131	382.25	-4.4356
388.02	6.1123	382.08	-4.4346
388.19	6.1153	381.92	-4.4327
388.35	6.1184	381.75	-4.4308
388.52	6.1144	381.58	-4.4319
388.69	6.1171	381.42	-4.4307
388.86	6.1246	381.25	-4.4312
389.02	6.1312	381.08	-4.4334
389.18	6.1322	380.92	-4.4362
389.35	6.1317	380.75	-4.4355
389.52	6.1324	380.59	-4.4301
389.69	6.1308	380.42	-4.4239
389.85	6.1303	380.26	-4.4227
390.02	6.1329	380.09	-4.4231
390.19	6.1372	379.92	-4.4248
390.35	6.1376	379.75	-4.4283
390.52	6.1434	379.59	-4.4285
390.68	6.1469	379.42	-4.4298
390.85	6.1469	379.25	-4.4335
391.02	6.15	379.09	-4.4345
391.19	6.1535	378.92	-4.4312
391.35	6.1544	378.75	-4.4299
391.52	6.1547	378.58	-4.4302
391.68	6.1568	378.42	-4.4281
391.85	6.1584	378.25	-4.4248
392.01	6.1603	378.08	-4.4255
392.18	6.1617	377.92	-4.4274
392.35	6.1636	377.75	-4.4288
392.52	6.1661	377.58	-4.4271
392.68	6.1706	377.42	-4.4268
392.85	6.1745	377.25	-4.4253
393.01	6.1776	377.08	-4.4244
393.18	6.1793	376.92	-4.4254
393.35	6.1808	376.75	-4.423
393.51	6.1835	376.59	-4.4235
393.68	6.1879	376.42	-4.4265
393.85	6.1886	376.25	-4.4242
394.01	6.1897	376.09	-4.4242
394.18	6.1927	375.92	-4.4261
394.35	6.1941	375.75	-4.425
394.51	6.1966	375.58	-4.4222
394.68	6.202	375.42	-4.4233
394.85	6.2024	375.26	-4.4246
395.01	6.2036	375.09	-4.4277
395.18	6.2046	374.92	-4.4314

195.47	5.1798	174.85	-4.352
195.63	5.1627	174.68	-4.3508
195.8	5.1472	174.52	-4.3492
195.96	5.1346	174.35	-4.3498
196.13	5.1212	174.18	-4.3511
196.3	5.1071	174.02	-4.3529
196.47	5.093	173.85	-4.3532
196.63	5.0812	173.68	-4.3539
196.8	5.0712	173.52	-4.3529
196.97	5.0619	173.35	-4.353
197.13	5.0525	173.18	-4.3527
197.3	5.0438	173.01	-4.352
197.46	5.0351	172.85	-4.3534
197.63	5.0244	172.68	-4.3544
197.8	5.0161	172.52	-4.3551
197.97	5.0103	172.35	-4.3538
198.13	5.0052	172.18	-4.3526
198.3	5.0014	172.01	-4.3528
198.47	4.998	171.85	-4.3534
198.63	4.9988	171.68	-4.3535
198.81	4.991	171.51	-4.3536
198.97	4.9891	171.34	-4.3543
199.14	4.9888	171.18	-4.3556
199.3	4.9876	171.01	-4.3536
199.47	4.9882	170.84	-4.3537
199.64	4.9892	170.68	-4.354
199.8	4.988	170.51	-4.3544
199.97	4.9846	170.35	-4.3539
200.14	4.981	170.18	-4.3547
200.3	4.9804	170.01	-4.355
200.47	4.9824	169.85	-4.3563
200.63	4.9836	169.68	-4.3568
200.8	4.9819	169.51	-4.3576
200.97	4.9822	169.35	-4.3593
201.14	4.9839	169.18	-4.3612
201.31	4.9837	169.01	-4.3627
201.47	4.9826	168.85	-4.3639
201.64	4.9835	168.68	-4.3662
201.81	4.9839	168.51	-4.3669
201.98	4.9843	168.34	-4.3677
202.14	4.9858	168.18	-4.3685
202.31	4.9866	168.01	-4.3684
202.47	4.9849	167.85	-4.3691
202.64	4.9825	167.68	-4.3694
202.81	4.9834	167.51	-4.3695
202.97	4.9844	167.34	-4.3715
203.14	4.9846	167.18	-4.3735
203.31	4.9854	167.01	-4.3746
203.48	4.9874	166.85	-4.3767
203.64	4.9873	166.68	-4.3802
203.81	4.9873	166.51	-4.3832

395.34	6.205	374.75	-4.4297
395.51	6.2096	374.59	-4.4289
395.68	6.2167	374.42	-4.4284
395.84	6.2187	374.26	-4.4281
396.01	6.2211	374.09	-4.4243
396.18	6.2279	373.92	-4.4227
396.34	6.2352	373.75	-4.4284
396.51	6.2361	373.59	-4.4352
396.68	6.2334	373.43	-4.4339
396.85	6.235	373.26	-4.431
397.01	6.2358	373.09	-4.427
397.18	6.2364	372.93	-4.4234
397.35	6.2429	372.76	-4.42
397.51	6.2468	372.59	-4.4186
397.68	6.2484	372.43	-4.4179
397.85	6.2517	372.26	-4.414
398.01	6.2556	372.1	-4.4167
398.18	6.2584	371.93	-4.4256
398.35	6.2604	371.76	-4.4338
398.52	6.2591	371.6	-4.4333
398.68	6.2595	371.43	-4.4306
398.85	6.2628	371.26	-4.4287
399.02	6.2658	371.1	-4.4284
399.18	6.2667	370.93	-4.4309
399.35	6.2672	370.76	-4.4317
399.51	6.2677	370.6	-4.4284
399.68	6.2675	370.43	-4.4266
399.85	6.2721	370.26	-4.4314
400.01	6.274	370.1	-4.4375
400.18	6.2753	369.93	-4.4032
400.35	6.2769	369.77	-4.3771
400.51	6.2783	369.6	-4.3852
400.68	6.2807	369.43	-4.4091
400.85	6.2805	369.27	-4.4596
401.01	6.279	369.1	-4.4761
401.18	6.2755	368.93	-4.4593
401.35	6.2726	368.77	-4.439
401.51	6.2714	368.6	-4.4289
401.68	6.2704	368.44	-4.424
401.85	6.2708	368.27	-4.4237
402.01	6.2697	368.1	-4.4246
402.18	6.2657	367.93	-4.4231
402.34	6.2592	367.76	-4.423
402.51	6.2497	367.6	-4.4245
402.68	6.2438	367.44	-4.4249
402.84	6.2409	367.27	-4.4254
403.01	6.235	367.1	-4.4267
403.18	6.2285	366.93	-4.4262
403.34	6.2223	366.77	-4.4265
403.51	6.2176	366.6	-4.4318
403.68	6.212	366.44	-4.4356

203.98	4.9888	166.34	-4.3861
204.14	4.9902	166.18	-4.3888
204.31	4.9919	166.01	-4.39
204.48	4.9926	165.84	-4.3937
204.65	4.9921	165.68	-4.3988
204.81	4.9913	165.51	-4.4023
204.98	4.991	165.34	-4.4061
205.15	4.9918	165.17	-4.4125
205.31	4.993	165	-4.4199
205.48	4.995	164.84	-4.4249
205.65	4.9954	164.67	-4.4315
205.81	4.9934	164.51	-4.4368
205.98	4.9926	164.34	-4.4441
206.15	4.9933	164.17	-4.4531
206.32	4.9973	164.01	-4.462
206.48	4.9982	163.84	-4.4693
206.64	4.9985	163.67	-4.4787
206.81	4.9966	163.5	-4.4889
206.98	4.9958	163.34	-4.4995
207.15	4.9974	163.17	-4.5124
207.31	4.9993	163.01	-4.5245
207.48	5.0012	162.84	-4.5362
207.64	5.0024	162.67	-4.5507
207.81	5.0041	162.5	-4.5658
207.98	5.0057	162.33	-4.5827
208.14	5.0066	162.17	-4.6014
208.31	5.0066	162.01	-4.6207
208.48	5.0081	161.84	-4.6399
208.65	5.0099	161.67	-4.6613
208.81	5.0109	161.5	-4.6853
208.98	5.0129	161.34	-4.7096
209.14	5.0141	161.17	-4.7353
209.31	5.0147	161.01	-4.7636
209.48	5.0144	160.84	-4.7962
209.65	5.0146	160.67	-4.8293
209.82	5.0149	160.5	-4.8602
209.98	5.0154	160.33	-4.8914
210.15	5.0169	160.16	-4.9265
210.32	5.0162	160	-4.9633
210.48	5.0153	159.83	-5.0009
210.65	5.0154	159.67	-5.0421
210.82	5.0157	159.5	-5.0842
210.98	5.0165	159.34	-5.1256
211.15	5.0176	159.17	-5.1684
211.32	5.0182	159	-5.2116
211.48	5.0171	158.83	-5.2549
211.65	5.0197	158.67	-5.2978
211.82	5.0231	158.5	-5.339
211.98	5.0254	158.34	-5.3795
212.15	5.0261	158.17	-5.4161
212.32	5.0283	158	-5.4466

403.84	6.1997	366.27	-4.4363
404.01	6.1921	366.1	-4.4325
404.18	6.1887	365.93	-4.4307
404.35	6.1857	365.77	-4.4313
404.51	6.1836	365.6	-4.4314
404.68	6.1771	365.43	-4.4314
404.84	6.1667	365.27	-4.4321
405.01	6.164	365.1	-4.434
405.18	6.1644	364.94	-4.4326
405.34	6.1632	364.77	-4.4321
405.51	6.1544	364.6	-4.4333
405.68	6.1454	364.44	-4.4333
405.84	6.1392	364.27	-4.4308
406.01	6.1307	364.11	-4.4308
406.18	6.1295	363.94	-4.4309
406.34	6.1254	363.78	-4.4303
406.51	6.1209	363.61	-4.4296
406.68	6.1177	363.44	-4.4319
406.84	6.1177	363.28	-4.4339
407.01	6.12	363.11	-4.434
407.17	6.1178	362.94	-4.4343
407.34	6.118	362.78	-4.437
407.51	6.1155	362.61	-4.4373
407.67	6.1153	362.44	-4.4364
407.84	6.1137	362.27	-4.4366
408.01	6.1087	362.11	-4.4388
408.18	6.1057	361.94	-4.4387
408.34	6.1067	361.77	-4.4384
408.51	6.1095	361.61	-4.4377
408.67	6.1123	361.44	-4.4353
408.84	6.1113	361.28	-4.4351
409.01	6.1107	361.11	-4.4359
409.18	6.1077	360.94	-4.4354
409.35	6.1052	360.77	-4.4332
409.51	6.1044	360.61	-4.4315
409.68	6.0988	360.44	-4.4327
409.84	6.0949	360.28	-4.431
410.01	6.0928	360.11	-4.4273
410.18	6.0928	359.94	-4.4115
410.34	6.0963	359.77	-4.3743
410.51	6.1005	359.61	-4.3739
410.68	6.1037	359.44	-4.3956
410.84	6.104	359.28	-4.4148
411.01	6.1007	359.11	-4.4232
411.17	6.0945	358.94	-4.4281
411.34	6.0963	358.78	-4.4514
411.51	6.0976	358.61	-4.4883
411.68	6.1	358.44	-4.4656
411.84	6.1023	358.28	-4.4318
412.01	6.1039	358.11	-4.4188
412.17	6.1031	357.95	-4.4191

212.48	5.0275	157.83	-5.4694
212.65	5.0283	157.67	-5.4852
212.82	5.029	157.5	-5.4937
212.98	5.0311	157.34	-5.4928
213.15	5.0338	157.17	-5.4815
213.32	5.0352	157	-5.4633
213.49	5.034	156.83	-5.4375
213.65	5.0343	156.66	-5.4024
213.82	5.0359	156.5	-5.3578
213.98	5.0372	156.33	-5.3059
214.15	5.0389	156.16	-5.249
214.32	5.0405	156	-5.1888
214.49	5.0379	155.83	-5.125
214.66	5.0385	155.66	-5.0618
214.82	5.0397	155.49	-4.9967
214.99	5.0411	155.32	-4.932
215.16	5.0422	155.16	-4.869
215.32	5.0436	154.99	-4.8094
215.49	5.0423	154.82	-4.7532
215.66	5.0396	154.65	-4.7019
215.83	5.0387	154.49	-4.6562
215.99	5.0414	154.32	-4.6147
216.16	5.0458	154.15	-4.5765
216.33	5.0508	153.99	-4.5434
216.49	5.0513	153.82	-4.5141
216.66	5.0513	153.65	-4.4878
216.82	5.0519	153.49	-4.4647
216.99	5.0523	153.32	-4.4451
217.16	5.0532	153.15	-4.429
217.32	5.0548	152.98	-4.4149
217.49	5.057	152.82	-4.4005
217.66	5.0592	152.65	-4.3885
217.82	5.0622	152.48	-4.3804
217.99	5.0625	152.32	-4.3722
218.16	5.0608	152.15	-4.3659
218.32	5.0587	151.98	-4.3615
218.49	5.0585	151.82	-4.3571
218.66	5.0612	151.65	-4.352
218.82	5.0608	151.48	-4.3471
218.99	5.0595	151.32	-4.345
219.16	5.0607	151.15	-4.3436
219.33	5.0617	150.98	-4.3415
219.49	5.0616	150.82	-4.3392
219.66	5.0613	150.65	-4.3367
219.82	5.0641	150.49	-4.354
219.99	5.0658	150.35	-4.3636
220.16	5.0672	150.26	-4.3846
220.33	5.0702	150.21	-4.3652
220.5	5.0718	150.17	-4.1829
220.67	5.0724	150.15	-3.7969
220.83	5.0735	150.14	-3.2775

412.34	6.1005	357.78	-4.4221
412.51	6.0982	357.61	-4.4274
412.67	6.0985	357.44	-4.4294
412.84	6.1006	357.28	-4.4241
413.01	6.1028	357.11	-4.417
413.17	6.1057	356.94	-4.4176
413.34	6.106	356.77	-4.4237
413.51	6.1026	356.61	-4.431
413.67	6.1016	356.44	-4.4346
413.84	6.107	356.27	-4.4399
414.01	6.1116	356.11	-4.4492
414.17	6.1137	355.94	-4.4495
414.33	6.1139	355.77	-4.4485
414.5	6.1098	355.61	-4.4691
414.67	6.1053	355.44	-4.4768
414.84	6.1041	355.27	-4.4655
415.01	6.1065	355.11	-4.4501
415.17	6.1092	354.94	-4.4405
415.34	6.1118	354.77	-4.4386
415.5	6.1104	354.61	-4.4367
415.67	6.108	354.44	-4.4357
415.84	6.1076	354.28	-4.438
416	6.109	354.11	-4.4398
416.17	6.1096	353.95	-4.4395
416.34	6.1081	353.78	-4.4398
416.5	6.1072	353.62	-4.4399
416.67	6.108	353.45	-4.4383
416.83	6.1103	353.29	-4.4394
417	6.1149	353.12	-4.4413
417.17	6.1163	352.95	-4.4425
417.34	6.1147	352.79	-4.4409
417.5	6.1155	352.62	-4.4414
417.67	6.1156	352.45	-4.4432
417.83	6.1123	352.28	-4.446
418	6.1163	352.12	-4.4448
418.17	6.1194	351.95	-4.4447
418.33	6.1195	351.78	-4.445
418.5	6.1174	351.61	-4.4514
418.67	6.1151	351.44	-4.5111
418.84	6.1166	351.28	-4.5183
419	6.1175	351.11	-4.48
419.17	6.1184	350.95	-4.3866
419.33	6.1215	350.78	-4.3689
419.5	6.1236	350.61	-4.3969
419.67	6.1214	350.45	-4.4385
419.83	6.1205	350.31	-4.4859
420	6.1211	350.22	-4.5256
420.17	6.1201	350.16	-4.4693
420.34	6.1187	350.13	-4.1909
420.5	6.1167	350.11	-3.6691
420.67	6.1155	350.1	-2.9937

220.99	5.074	150.13	-2.7081
221.16	5.0736	150.13	-2.1542
221.33	5.0765	150.13	-1.66
221.5	5.0777	150.13	-1.2425
221.66	5.0789	150.13	-0.9051
221.83	5.0795	150.13	-0.6391
222	5.0813	150.13	-0.4341
222.16	5.0814	150.13	-0.2787
222.33	5.082	150.13	-0.16
222.5	5.0827	150.13	-0.0689
222.66	5.0819	150.13	-0.0016
222.83	5.0815	150.13	0.0458
223	5.0812	150.13	0.0804
223.16	5.0828	150.13	0.1056
223.33	5.0831	150.13	0.1238
223.5	5.0834	150.14	0.1367
223.66	5.084	150.13	0.1457
223.83	5.0858	150.14	0.1516
224	5.0886	150.13	0.1567
224.17	5.0906	150.13	0.1612
224.33	5.0926	150.13	0.1643
224.5	5.0944	150.13	0.1665
224.66	5.0955	150.13	0.1672
224.83	5.0981	150.13	0.1677
225	5.0995	150.13	0.1685
225.17	5.0998	150.14	0.17
225.34	5.1014	150.14	0.17
225.5	5.1025	150.13	0.1685
225.66	5.103	150.14	0.168
225.83	5.1043	150.13	0.1678
226	5.1055	150.13	0.1681
226.17	5.1053	150.13	0.1692
226.34	5.107	150.14	0.1706
226.51	5.107	150.13	0.1697
226.67	5.1079	150.14	0.1695
226.84	5.1098	150.13	0.1696
227	5.1091	150.13	0.1687
227.17	5.1091	150.13	0.169
227.34	5.1105	150.13	0.1698
227.51	5.1126	150.13	0.1692
227.67	5.1133	150.13	0.1691
227.84	5.1141	150.13	0.1694
228	5.1166	150.14	0.1697
228.16	5.1174	150.13	0.17
228.33	5.1188	150.14	0.1707
228.5	5.1189	150.13	0.1704
228.67	5.1191	150.13	0.1693
228.83	5.1181	150.14	0.169
229	5.1194	150.13	0.1701
229.17	5.1215	150.13	0.171
229.33	5.1228	150.14	0.1711

420.84	6.1157	350.09	-2.2887
421	6.1179	350.09	-1.6423
421.17	6.1198	350.09	-1.1016
421.34	6.1219	350.09	-0.6734
421.5	6.1243	350.09	-0.3446
421.67	6.1282	350.09	-0.0935
421.84	6.1338	350.09	0.0928
422	6.1356	350.09	0.233
422.17	6.1321	350.09	0.3388
422.34	6.1301	350.09	0.4135
422.5	6.1314	350.09	0.4703
422.67	6.1333	350.09	0.5112
422.83	6.1339	350.09	0.5379
423	6.1367	350.09	0.5616
423.17	6.1378	350.08	0.5792
423.34	6.1374	350.09	0.5908
423.5	6.1343	350.09	0.5999
423.67	6.1362	350.08	0.6051
423.83	6.1401	350.08	0.6093
424	6.1412	350.08	0.6137
424.17	6.1384	350.09	0.616
424.33	6.1375	350.08	0.6175
424.5	6.1372	350.08	0.619
424.67	6.141	350.08	0.6188
424.83	6.1438	350.08	0.6178
425	6.1406	350.08	0.6197
425.16	6.1396	350.08	0.6231
425.33	6.141	350.08	0.6232
425.5	6.1437	350.08	0.621
425.66	6.1463	350.08	0.6197
425.83	6.1456	350.08	0.6206
426	6.1451	350.08	0.6206
426.16	6.1476	350.09	0.6231
426.33	6.1504	350.09	0.6219
426.5	6.1522	350.08	0.6205
426.66	6.1495	350.08	0.6233
426.83	6.146	350.08	0.625
427	6.1429	350.08	0.6243
427.17	6.1491	350.09	0.6215
427.33	6.1527	350.08	0.6207
427.5	6.1546	350.08	0.622
427.66	6.1563	350.08	0.6226
427.83	6.1582	350.08	0.623
428	6.1557	350.08	0.624
428.17	6.1563	350.09	0.6219
428.33	6.1574	350.09	0.6218
428.5	6.1551	350.08	0.6227
428.67	6.1566	350.09	0.6225
428.83	6.1523	350.08	0.6234
429	6.1458	350.08	0.6243
429.17	6.1481	350.08	0.6245

229.5	5.1252	150.13	0.1706
229.67	5.1276	150.13	0.1707
229.84	5.13	150.14	0.1718
230	5.1302	150.14	0.1721
230.17	5.1295	150.14	0.1721
230.33	5.1297	150.13	0.1725
230.5	5.1292	150.13	0.173
230.67	5.1294	150.13	0.1722
230.84	5.1308	150.13	0.1721
231	5.1309	150.13	0.1717
231.17	5.1314	150.13	0.1724
231.34	5.1337	150.14	0.1711
231.5	5.1352	150.13	0.1701
231.67	5.1337	150.13	0.1712
231.84	5.1322	150.13	0.1718
232.01	5.1325	150.13	0.172
232.17	5.1351	150.13	0.1721
232.34	5.1364	150.13	0.1724
232.51	5.1372	150.13	0.1721
232.67	5.137	150.13	0.1714
232.84	5.1374	150.13	0.1712
233.01	5.1384	150.14	0.1712
233.18	5.1393	150.14	0.1719
233.34	5.1417	150.14	0.1725
233.51	5.1439	150.14	0.1727
233.67	5.1448	150.14	0.1722
233.84	5.1461	150.14	0.1706
234.01	5.1492	150.14	0.1709
234.17	5.1502	150.13	0.1729
234.34	5.1509	150.14	0.1744
234.51	5.1528	150.13	0.1737
234.68	5.1535	150.13	0.1733
234.84	5.1548	150.13	0.1726
235.01	5.1576	150.13	0.1716
235.17	5.1597	150.13	0.172
235.34	5.159	150.13	0.1724
235.5	5.1594	150.13	0.173
235.67	5.1599	150.14	0.1727
235.84	5.1593	150.14	0.1728
236.01	5.1577	150.14	0.1724
236.17	5.1565	150.14	0.1714
236.34	5.1563	150.14	0.1712
236.5	5.158	150.14	0.1714
236.67	5.1599	150.14	0.1716
236.84	5.1635	150.14	0.1722
237.01	5.1682	150.14	0.1725
237.18	5.1709	150.14	0.1722
237.34	5.1723	150.14	0.1726
237.51	5.1719	150.14	0.1728
237.68	5.1712	150.13	0.1722
237.84	5.1714	150.14	0.1728

429.33	6.1536	350.08	0.6242
429.5	6.1602	350.08	0.6243
429.67	6.1632	350.08	0.6222
429.83	6.1644	350.08	0.6187
430	6.1678	350.08	0.6199
430.17	6.1718	350.08	0.6218
430.33	6.1762	350.08	0.6229
430.5	6.1773	350.08	0.6225
430.67	6.1808	350.08	0.6192
430.84	6.1836	350.08	0.6206
431	6.183	350.08	0.6231
431.17	6.1836	350.08	0.6236
431.34	6.1832	350.09	0.623
431.51	6.1814	350.08	0.6251
431.68	6.1821	350.08	0.625
431.84	6.1846	350.09	0.6227
432.01	6.1871	350.08	0.6212
432.17	6.1859	350.08	0.6213
432.34	6.19	350.08	0.6211
432.5	6.1973	350.08	0.6201
432.67	6.201	350.08	0.621
432.84	6.1943	350.08	0.6195
433	6.1898	350.08	0.6185
433.17	6.1902	350.08	0.6169
433.34	6.19	350.08	0.6176
433.5	6.1895	350.08	0.6212
433.67	6.1885	350.08	0.6222
433.84	6.1932	350.08	0.6219
434.01	6.1905	350.08	0.6212
434.17	6.1897	350.08	0.6221
434.34	6.19	350.09	0.6233
434.51	6.1913	350.09	0.6223
434.67	6.1935	350.08	0.6208
434.83	6.1994	350.09	0.62
435	6.1988	350.08	0.6184
435.17	6.1961	350.08	0.6198
435.34	6.191	350.08	0.622
435.5	6.1853	350.08	0.6225
435.67	6.189	350.08	0.6209
435.83	6.1962	350.08	0.6204
436	6.2023	350.08	0.6177
436.17	6.203	350.08	0.6186
436.33	6.2012	350.08	0.6222
436.5	6.2001	350.08	0.621
436.67	6.2031	350.08	0.6177
436.84	6.2104	350.08	0.6164
437	6.2162	350.08	0.6158
437.17	6.218	350.08	0.6175
437.34	6.2134	350.08	0.6194
437.5	6.207	350.08	0.6196
437.67	6.2038	350.08	0.6203

238.01	5.1735	150.14	0.174
238.17	5.1746	150.14	0.1739
238.34	5.1752	150.14	0.172
238.51	5.1771	150.14	0.1703
238.67	5.1775	150.14	0.1705
238.84	5.1786	150.14	0.171
239.01	5.1788	150.14	0.1716
239.17	5.1821	150.14	0.1719
239.34	5.186	150.14	0.1727
239.51	5.1891	150.14	0.1708
239.68	5.1888	150.14	0.1692
239.84	5.1863	150.14	0.1685
240.01	5.1893	150.14	0.1691
240.17	5.1926	150.14	0.1697
240.34	5.1932	150.14	0.1708
240.51	5.1923	150.14	0.1709
240.68	5.1908	150.14	0.1707
240.84	5.1895	150.14	0.1707
241.01	5.1898	150.14	0.1697
241.17	5.1913	150.14	0.1683
241.34	5.1921	150.14	0.1688
241.51	5.1945	150.14	0.1691
241.68	5.1974	150.14	0.1681
241.85	5.1992	150.14	0.1682
242.01	5.1994	150.14	0.1675
242.18	5.2005	150.13	0.1677
242.35	5.2018	150.14	0.1688
242.51	5.2035	150.14	0.1692
242.68	5.2053	150.14	0.1677
242.85	5.2032	150.14	0.1669
243.01	5.2031	150.14	0.1662
243.18	5.2048	150.14	0.1658
243.35	5.205	150.14	0.1654
243.51	5.2058	150.14	0.1663
243.68	5.2074	150.14	0.1663
243.85	5.2081	150.14	0.1665
244.01	5.2101	150.14	0.1675
244.18	5.2114	150.13	0.1663
244.35	5.2123	150.14	0.1654
244.52	5.2122	150.14	0.1656
244.68	5.2128	150.14	0.1658
244.85	5.2129	150.14	0.1657
245.01	5.2133	150.14	0.1647
245.18	5.2153	150.14	0.1637
245.35	5.2153	150.14	0.1647
245.51	5.2153	150.14	0.1656
245.68	5.2185	150.14	0.1655
245.85	5.2191	150.14	0.1662
246.01	5.2169	150.14	0.166
246.18	5.217	150.13	0.1651
246.35	5.221	150.14	0.1637

437.84	6.2052	350.08	0.6196
438.01	6.2036	350.08	0.6181
438.17	6.2061	350.08	0.6159
438.34	6.211	350.08	0.6163
438.5	6.2146	350.08	0.6162
438.67	6.2155	350.08	0.6156
438.84	6.2148	350.08	0.618
439	6.2143	350.08	0.619
439.17	6.2157	350.08	0.6184
439.34	6.2188	350.08	0.6195
439.5	6.2173	350.08	0.6191
439.67	6.2169	350.08	0.6161
439.84	6.2177	350.08	0.6156
440.01	6.2156	350.08	0.6146
440.17	6.2174	350.08	0.6165
440.34	6.2213	350.08	0.6196
440.5	6.2215	350.08	0.6193
440.67	6.2213	350.08	0.6174
440.84	6.2211	350.08	0.6145
441	6.2194	350.08	0.6132
441.17	6.2182	350.08	0.6128
441.34	6.2177	350.08	0.6124
441.51	6.2179	350.08	0.6133
441.67	6.2223	350.08	0.615
441.83	6.2223	350.08	0.6157
442	6.2254	350.08	0.6152
442.17	6.2279	350.08	0.6139
442.34	6.2265	350.08	0.6137
442.5	6.2237	350.08	0.6148
442.67	6.2268	350.08	0.6158
442.84	6.2295	350.08	0.6166
443	6.2292	350.08	0.6156
443.17	6.2312	350.08	0.6151
443.34	6.2341	350.08	0.6143
443.5	6.2385	350.08	0.6133
443.67	6.2417	350.08	0.6126
443.84	6.243	350.08	0.6135
444.01	6.2463	350.08	0.6132
444.17	6.2487	350.08	0.615
444.34	6.2476	350.08	0.6144
444.5	6.2451	350.08	0.6122
444.67	6.2436	350.08	0.6098
444.84	6.2438	350.08	0.6112
445.01	6.2471	350.08	0.6131
445.17	6.2511	350.08	0.6136
445.33	6.2543	350.08	0.6136
445.5	6.2453	350.08	0.6136
445.67	6.2379	350.08	0.613
445.83	6.2406	350.08	0.6124
446	6.2459	350.08	0.6115
446.17	6.2525	350.08	0.609

246.51	5.2239	150.14	0.1624
246.68	5.2229	150.14	0.1628
246.85	5.2247	150.14	0.1635
247.01	5.2285	150.14	0.1642
247.17	5.2303	150.14	0.164
247.34	5.2276	150.14	0.1641
247.51	5.2263	150.14	0.1631
247.68	5.2269	150.13	0.162
247.84	5.229	150.14	0.1624
248.02	5.2314	150.14	0.1623
248.17	5.2332	150.13	0.1616
248.34	5.2327	150.14	0.1614
248.51	5.2311	150.13	0.1615
248.68	5.2294	150.14	0.1614
248.85	5.2279	150.14	0.162
249.01	5.2284	150.14	0.1618
249.18	5.2329	150.14	0.1617
249.35	5.2369	150.14	0.1618
249.51	5.2388	150.14	0.1616
249.68	5.238		
249.84	5.2149		
249.98	5.1928		
250.06	5.2261		
250.1	5.1836		
250.12	4.918		
250.14	4.4076		
250.13	3.7468		
250.13	3.0578		
250.13	2.4297		
250.13	1.9042		
250.12	1.489		
250.12	1.175		
250.12	0.9439		
250.12	0.775		
250.11	0.6544		
250.11	0.5688		
250.11	0.5102		
250.11	0.4675		
250.11	0.4381		
250.11	0.416		
250.11	0.4063		
250.11	0.4026		
250.11	0.4022		
250.11	0.4021		
250.11	0.4001		
250.11	0.3982		
250.11	0.3978		
250.11	0.3988		
250.11	0.3985		
250.11	0.3996		
250.11	0.3978		

446.33	6.2544	350.08	0.6087
446.49	6.2561	350.08	0.6102
446.66	6.258	350.09	0.6127
446.83	6.264	350.09	0.6139
447	6.2663	350.09	0.6104
447.16	6.2645	350.09	0.6096
447.33	6.2642	350.09	0.6101
447.5	6.2626	350.09	0.6116
447.67	6.2623	350.09	0.6136
447.83	6.2595	350.09	0.6145
448	6.2602	350.08	0.6143
448.17	6.2646	350.08	0.6117
448.34	6.2663	350.08	0.6099
448.5	6.2669	350.08	0.6097
448.67	6.2707	350.08	0.6093
448.84	6.2712	350.08	0.6104
449	6.2716	350.08	0.6115
449.17	6.2719	350.08	0.6116
449.33	6.2714	350.08	0.6126
449.5	6.2743		
449.67	6.2607		
449.8	6.2553		
449.89	6.2965		
449.94	6.2372		
449.97	5.9059		
449.99	5.3027		
449.99	4.5581		
450	3.809		
450	3.1545		
450	2.6276		
450	2.2282		
450	1.9296		
450	1.7111		
450	1.5522		
450.01	1.4419		
450	1.3644		
450	1.3049		
450	1.2601		
450	1.2294		
450	1.207		
450	1.1887		
450	1.175		
450	1.1726		
450	1.1683		
450	1.164		
450	1.1617		
450.01	1.1574		
450	1.1535		
450	1.1528		
450	1.1536		
450	1.1515		

250.11	0.3979		
250.11	0.3988		
250.11	0.4006		
250.11	0.4022		
250.11	0.4011		
250.11	0.3993		
250.11	0.3991		
250.11	0.3998		
250.11	0.4012		
250.11	0.4026		
250.11	0.4026		
250.11	0.4027		
250.11	0.4024		
250.11	0.4012		
250.11	0.4015		
250.11	0.4012		
250.11	0.4007		
250.11	0.3999		
250.11	0.4012		
250.11	0.4023		
250.11	0.4032		
250.11	0.4018		
250.11	0.3993		
250.11	0.3994		
250.11	0.4005		
250.11	0.4024		
250.11	0.4027		
250.11	0.4031		
250.11	0.4027		
250.11	0.3999		
250.11	0.3986		
250.11	0.3983		
250.11	0.3993		
250.11	0.4006		
250.11	0.3998		
250.11	0.4006		
250.11	0.4018		
250.11	0.402		
250.11	0.4023		
250.11	0.4006		
250.11	0.3999		
250.11	0.4006		
250.11	0.401		
250.11	0.4018		
250.11	0.4039		
250.11	0.404		
250.11	0.4028		
250.11	0.402		
250.11	0.4024		
250.11	0.4021		
250.11	0.4012		

450	1.1496		
450	1.1511		
450	1.1501		
450.01	1.1497		
450	1.1513		
450	1.1521		
450	1.1513		
450	1.1529		
450	1.1516		
450	1.1499		
450	1.1497		
450.01	1.1482		
450	1.1442		
450.01	1.1489		
450	1.1495		
450	1.1465		
450.01	1.1485		
450	1.1469		
450	1.1471		
450.01	1.1486		
450	1.1494		
450	1.1482		
450	1.1474		
450.01	1.1402		
450.01	1.1327		
450	1.1331		
450.01	1.1446		
450	1.1477		
450.01	1.1474		
450	1.1489		
450	1.1509		
450.01	1.1533		
450	1.1562		
450	1.1572		
450	1.157		
450.01	1.1537		
450.01	1.1486		
450.01	1.1448		
450	1.1436		
450	1.1429		
450.01	1.1464		
450.01	1.151		
450.01	1.1482		
450.01	1.1471		
450.01	1.1487		
450.01	1.1487		
450.01	1.1457		
450.01	1.1448		
450.01	1.1447		
450.01	1.1469		
450.01	1.1465		

250.11	0.4011		
250.11	0.4021		
250.11	0.4025		
250.11	0.4031		
250.11	0.4021		
250.11	0.4013		
250.11	0.3999		
250.11	0.4009		
250.11	0.4007		
250.11	0.4016		
250.11	0.4001		
250.11	0.3974		
250.11	0.3974		
250.11	0.4017		
250.11	0.4045		
250.11	0.4023		
250.11	0.4		
250.11	0.3997		
250.11	0.402		
250.11	0.4024		
250.11	0.4022		
250.11	0.4023		
250.11	0.4022		
250.11	0.3999		
250.11	0.3987		
250.11	0.4003		
250.11	0.4018		
250.11	0.4024		
250.11	0.4018		
250.11	0.4015		
250.11	0.4017		
250.11	0.4016		
250.11	0.4006		
250.11	0.4009		
250.11	0.4009		
250.11	0.4012		
250.11	0.4013		
250.11	0.3995		
250.11	0.3997		
250.11	0.4003		
250.11	0.401		
250.11	0.4015		
250.11	0.4014		
250.11	0.4018		
250.11	0.4005		
250.11	0.4012		
250.11	0.4027		
250.11	0.4023		
250.11	0.4018		
250.11	0.4009		
250.11	0.4015		

450.01	1.1457		
450.01	1.1444		
450.01	1.1459		
450	1.1466		
450.01	1.1433		
450.01	1.1458		
450.01	1.1456		
450.01	1.1424		
450.01	1.1432		
450.01	1.1416		
450.01	1.1424		
450.01	1.146		
450.01	1.1479		
450.01	1.1454		
450.01	1.1412		
450.01	1.1401		
450.01	1.14		
450.01	1.1403		
450.01	1.142		
450.01	1.1461		
450.01	1.1445		
450.01	1.1406		
450.01	1.1333		
450.01	1.1383		
450.01	1.1438		
450.01	1.1454		
450.01	1.1444		
450.01	1.1449		
450.01	1.145		
450.01	1.1411		
450.01	1.1378		
450.01	1.1381		
450.01	1.1355		
450.01	1.1351		
450.01	1.1371		
450.01	1.1401		
450.01	1.1436		
450.01	1.1428		
450.01	1.1433		
450.01	1.1385		
450.01	1.1378		
450.01	1.1376		
450.01	1.1391		
450.01	1.138		
450.01	1.1382		
450.01	1.1389		
450.01	1.1391		
450.01	1.1401		
450.01	1.1393		
450.01	1.1367		
450.01	1.1392		

250.11	0.4022		
250.11	0.4031		
250.11	0.402		
250.11	0.4016		
250.11	0.402		
250.11	0.4022		
250.11	0.4035		
250.11	0.4046		
250.11	0.4037		
250.11	0.4011		
250.11	0.4006		
250.11	0.4015		
250.11	0.4014		
250.11	0.4018		
250.11	0.4006		
250.11	0.3988		
250.11	0.3985		
250.11	0.3996		
250.11	0.4015		
250.11	0.4031		
250.11	0.4042		
250.11	0.4051		
250.11	0.4036		
250.11	0.4016		
250.11	0.4009		
250.11	0.3994		
250.11	0.3987		
250.11	0.3996		
250.11	0.4008		
250.11	0.403		
250.11	0.4019		
250.11	0.4007		
250.11	0.3996		
250.11	0.398		
250.11	0.3985		
250.11	0.3984		
250.11	0.3989		
250.11	0.4012		
250.11	0.4002		
250.11	0.4		
250.11	0.4008		
250.11	0.4004		
250.11	0.4014		
250.11	0.4021		
250.11	0.4012		
250.11	0.3998		
250.11	0.3986		
250.11	0.398		

450.01	1.1407		
450.01	1.1402		
450.01	1.1404		
450.01	1.1394		
450.01	1.133		
450.01	1.1329		
450.01	1.1397		
450.01	1.1401		
450.01	1.1388		
450.01	1.1376		
450.01	1.1357		
450.01	1.1337		
450.01	1.1361		
450.01	1.1342		
450.01	1.1348		
450.01	1.1373		
450.01	1.1394		
450.01	1.1399		
450.01	1.1412		
450.01	1.1386		
450.01	1.1375		
450.01	1.1371		
450.01	1.1369		
450.01	1.1372		
450.01	1.1396		
450.01	1.1367		
450.01	1.1355		
450.01	1.1348		
450.01	1.133		
450.01	1.1266		
450.01	1.1273		
450.01	1.1316		
450.01	1.1373		
450.01	1.1409		
450.01	1.1418		
450.01	1.1385		
450.01	1.1374		
450.01	1.136		
450.01	1.138		
450.01	1.1396		
450.01	1.1419		
450.01	1.1376		
450.01	1.1325		
450.01	1.1326		
450.01	1.1313		
450.01	1.1343		
450.01	1.1373		
450.01	1.1375		
