# Supplementary material for: Investigations on the polymorphism of K4CaSi6O15 at elevated temperatures
Source: J Am Ceram Soc. 2023 Jul 13;106(11):7109–22. doi: 10.1111/jace.19310 (PMC10962641; doi:10.1111/jace.19310)
Supplement: Supplementary file 4 — Supporting information [file JACE-106-7109-s003.pdf]

Table S3. Displacements for each atom pairing after transformation of the high temperature structure in a subgroup basis.

Atom	Atomic Displacements			
	U_x	U_y	U_z	$\|u\|$
Ca(1)_2 - Ca(1)*	0.0372	0.0011	-0.0153	0.3292
Ca(1)_3 - Ca(2)	-0.005	0	0.0052	0.0748
Ca(1) - Ca(3)	-0.0314	0.0011	0.008	0.2463
Si(2) - Si(1)	-0.0214	0.0015	0.0093	0.1975
Si(2)_5 - Si(2)	-0.0053	0.0037	0.0042	0.1205
Si(1)_5 - Si(3)	0.0357	0.0015	-0.0132	0.3065
Si(2)_2 - Si(4)	0.0371	-0.0019	-0.0131	0.3158
Si(3)_5 - Si(5)	-0.0009	0.0009	-0.0016	0.0307
Si(3)_2 - Si(6)	-0.0096	-0.0015	0.0015	0.0814
Si(3) - Si(7)	0.0328	0.0009	-0.0048	0.2395
Si(2)_3 - Si(8)	-0.0359	-0.0033	0.0044	0.2734
Si(2)_4 - Si(9)	0.0479	-0.0034	-0.0104	0.3744
Si(1)_2 - Si(10)	-0.0049	0.004	0.0058	0.1369
Si(3)_6 - Si(11)	0.0339	0.0007	-0.0062	0.2521
Si(1)_4 - Si(12)	-0.0201	0.0065	-0.0006	0.2249
Si(1)_6 - Si(13)	-0.0338	-0.0049	0.0108	0.3077
Si(1)_3 - Si(14)	0.0327	0.0006	-0.0045	0.2371
Si(3)_3 - Si(15)	-0.0168	0.0044	0.001	0.1691
Si(3)_4 - Si(16)	-0.0382	-0.0002	0.0101	0.2996
Si(1) - Si(17)	-0.0081	-0.0029	0.0008	0.0973
Si(2)_6 - Si(18)	-0.0148	0.0022	0.0026	0.1245
O(3) - O(1)	-0.0085	-0.0013	0.0186	0.2415
O(7)_2 - O(2)	0.0639	0.0143	-0.0052	0.5967
O(3)_5 - O(3)	0.0326	0.0005	-0.0188	0.3324
O(8)_3 - O(4)	-0.0618	0.011	0.0101	0.5437
O(8)_6 - O(5)	-0.0341	0.01	0.0027	0.3651
O(6)_4 - O(6)	0.0383	-0.0031	-0.0086	0.3039
O(1)_3 - O(7)	-0.0056	0.0004	0.011	0.1432
O(4)_4 - O(8)	0.0663	-0.0123	-0.0224	0.6444
O(7)_4 - O(9)	0.0682	0.015	-0.0116	0.6489
O(4) - O(10)	-0.0393	-0.005	0.0099	0.3349
O(1)_2 - O(11)	0.0144	0.0046	-0.0047	0.1719
O(5) - O(12)	0.0501	-0.011	-0.0266	0.5754
O(8) - O(13)	-0.0283	0.0028	0.0118	0.2624
O(2)_5 - O(14)	-0.0048	-0.0031	0.0023	0.0961
O(4)_2 - O(15)	0.0489	-0.0081	-0.0193	0.4793
O(2) - O(16)	-0.0267	0.0032	0.0044	0.2152
O(6)_5 - O(17)	-0.0162	0.0015	0.0064	0.1473
O(3)_6 - O(18)	-0.0107	0.001	0.0152	0.2066
O(7)_3 - O(19)	-0.0536	0.0096	0.0183	0.5177
O(8)_2 - O(20)	0.0421	0.0025	-0.0177	0.3802
O(4)_6 - O(21)	-0.0192	-0.0027	0.0193	0.2881
O(2)_4 - O(22)	0.0365	0.0016	-0.011	0.2972
O(4)_3 - O(23)	-0.0774	-0.0236	0.0114	0.8572

O(2)_2 - O(24)	0.0288	-0.0017	-0.0075	0.2297
O(6)_2 - O(25)	0.041	-0.0009	-0.0142	0.343
O(7)_6 - O(26)	-0.0331	0.0154	0.0082	0.4923
O(1)_6 - O(27)	0.0225	-0.0074	0.0017	0.2556
O(1)_5 - O(28)	-0.0092	0.0004	-0.0053	0.0882
O(3)_3 - O(29)	0.0293	0.0001	-0.0293	0.4225
O(6) - O(30)	-0.0255	0.006	0.0085	0.2673
O(1) - O(31)	0.0197	-0.0036	-0.0091	0.2063
O(2)_6 - O(32)	-0.0029	-0.0004	0.0061	0.0796
O(4)_5 - O(33)	-0.023	0.0101	0.01	0.3447
O(7) - O(34)	-0.0503	0.0221	0.0059	0.7046
O(1)_4 - O(35)	0.0458	0.0081	-0.0203	0.4709
O(5)_3 - O(36)	-0.0376	-0.01	0.0324	0.5602
O(3)_4 - O(37)	-0.0306	-0.0039	-0.0062	0.2443
O(8)_5 - O(38)	-0.007	0.0014	0.0092	0.1318
O(5)_2 - O(39)	-0.024	-0.0106	0.0163	0.3942
O(2)_3 - O(40)	-0.05	-0.0026	0.0116	0.3896
O(6)_3 - O(41)	-0.0456	0.004	0.0098	0.3625
O(3)_2 - O(42)	0.0071	0.0013	-0.0098	0.1368
O(7)_5 - O(43)	-0.0343	0.0083	0.0035	0.3335
O(8)_4 - O(44)	0.0637	0.0066	-0.0135	0.5138
O(6)_6 - O(45)	-0.0033	-0.0014	-0.0028	0.0549
K(1)_5 - K(1)	0.0192	-0.0048	-0.0193	0.3076
K(1)_3 - K(2)	0.0029	-0.0033	-0.0231	0.2974
K(1)_2 - K(3)	-0.0042	-0.0018	0.0006	0.058
K(1)_6 - K(4)	-0.0018	-0.0017	0.0173	0.218
K(2)_3 - K(5)	0.0406	0.0078	-0.0039	0.3581
K(2)_4 - K(6)	-0.0388	0.0059	0.0008	0.3148
K(2) - K(7)	-0.0121	0.0045	0.0009	0.1498
K(1) - K(8)	0.0211	-0.0019	0.0127	0.2132
K(2)_5 - K(9)	0.0233	-0.0014	-0.0094	0.2073
K(2)_6 - K(10)	0.0063	-0.0073	0.0081	0.2261
K(2)_2 - K(11)	0.0083	-0.0018	0.0049	0.095
K(1)_4 - K(12)	-0.033	-0.0021	0.012	0.2857

*The first entry denotes the atomic sites in the high-symmetry structure, while the second refers to the corresponding atomic sites in the low-symmetry structure.
